# Quantitative Proteomic Analysis Reveals Yeast Cell Wall Products Influence the Serum Proteome Composition of Broiler Chickens

**DOI:** 10.3390/ijms231911844

**Published:** 2022-10-06

**Authors:** Niall Conlon, Richard A. Murphy, Aoife Corrigan, Sean Doyle, Rebecca A. Owens, Sheena Fagan

**Affiliations:** 1 Department of Biology, Maynooth University, Maynooth, W23 F2H6 Co. Kildare, Ireland; 2 Alltech Biosciences Centre, Sarney, Summerhill Road, Dunboyne, A86 X006 Co. Meath, Ireland

**Keywords:** gut health, poultry, quantitative proteomics, yeast cell wall, immunity, inflammation, intestinal integrity

## Abstract

With an ever-growing market and continual financial pressures associated with the prohibition of antibiotic growth promoters, the poultry industry has had to rapidly develop non-antibiotic alternatives to increase production yields. A possible alternative is yeast and its derivatives, such as the yeast cell wall (YCW), which have been proposed to confer selected beneficial effects on the host animal. Here, the effect of YCW supplementation on the broiler chicken was investigated using a quantitative proteomic strategy, whereby serum was obtained from three groups of broilers fed with distinct YCW-based Gut Health Products (GHP) or a control basal diet. Development of a novel reagent enabled application of ProteoMiner™ technology for sample preparation and subsequent comparative quantitative proteomic analysis revealed proteins which showed a significant change in abundance (*n* = 167 individual proteins; *p* < 0.05); as well as proteins which were uniquely identified (*n* = 52) in, or absent (*n* = 37) from, GHP-fed treatment groups versus controls. An average of 7.1% of proteins showed changes in abundance with GHP supplementation. Several effects of these GHPs including immunostimulation (via elevated complement protein detection), potential alterations in the oxidative status of the animal (e.g., glutathione peroxidase and catalase), stimulation of metabolic processes (e.g., differential abundance of glyceraldehyde-3-phosphate dehydrogenase), as well as evidence of a possible hepatoprotective effect (attenuated levels of serum α-glutathione s-transferase) by one GHP feed supplement, were observed. It is proposed that specific protein detection may be indicative of GHP efficacy to stimulate broiler immune status, i.e., may be biomarkers of GHP efficacy. In summary, this work has developed a novel technology for the preparation of high dynamic range proteomic samples for LC-MS/MS analysis, is part of the growing area of livestock proteomics and, importantly, provides evidential support for beneficial effects that GHP supplementation has on the broiler chicken.

## 1. Introduction

Consequent to bans on the use of antibiotic growth promoters (AGP) in animal husbandry, interest in alternative growth promoters has grown immensely. Yeast and its derivatives, such as the mannan-rich fraction (MRF) of the yeast cell wall (YCW), have emerged as viable alternatives to AGP and have been shown to impart several beneficial effects on animals and have application in the field of animal husbandry. Some noted effects of yeast-based products include modulation of the gut microbiome [1], reduced infection by pathogenic bacteria and beneficial stimulation of the host immune system [2,3,4,5,6,7,8].

Immunological responses triggered by the YCW are largely due to the action of two molecules, Mannose Oligosaccharide (MOS) and β-glucan which are highly abundant in YCW. YCW immunostimulation has been reported in a number of species including human [9], fish [7] and mice [10] as well as livestock such as cows [11], pigs [12,13] and broiler chickens [4] and has been shown to induce a range of immunological effects both in vivo and in vitro. These benefits include reducing the levels of pathogenic bacteria in the gut [14], enhancing growth of beneficial bacteria [15] and modulating the immune response [16]. MOS have been found to modulate the gut microbiome by acting as high affinity ligands offering competitive binding sites for mannose-specific type-1 fimbriae of pathogenic bacteria [17] and stimulating the production of mucins by goblet cells found in the digestive tract [14]. Mucins are the glycoprotein constituent of mucus [18] which are involved in the binding and clearance of bacteria from the intestine. This reduction in pathogenic bacteria can create a more favourable environment for beneficial bacteria such as Lactobacillus and Bifidobacterium spp. in the broiler intestine [19]. Thus, intestinal homeostasis depends on complex interactions between microbiota, intestinal epithelium, and host immune system [1].

The physiological effects of MOS can translate into improved performance of broilers. Several studies have suggested that birds fed MOS as a supplement to a basal diet had significantly improved weight gain when compared to those fed the basal diet alone [20,21]. A review of the different mechanisms of MOS and their subsequent effect on intestinal microflora, intestinal morphology, immunity, and performance of broilers are discussed in detail elsewhere [14]. With developments in mass spectrometry and bioinformatic technologies in the last decade [22], comparative proteomics has come to the forefront of proteomic research, including a number of reviews published highlighting its potential application in farm animal research [23,24,25]. However, despite advances in proteomic technologies, only a relatively small number of comparative proteomic studies have been conducted on avian serum in recent years [26,27,28,29,30,31]. 

Packialakshmi et al. revealed differential protein profiles among low abundance proteins in chicken plasma, following depletion of high abundant proteins, in an LPS challenge versus control study (*n* = 9 birds/group) using both LC-and MALDI-MS/MS analyses [32]. Chemokine CCLI10 and alpha 1 acid glycoprotein (A1AGP) were only detected in plasma from LPS-treated birds. Label-free proteomic analysis identified increased abundance of A1AGP, CATH-2 precursor, a heparinase precursor protein and reduced abundance of ISG12-2 in plasma following LPS-only exposure, leading the authors to propose the utility of plasma proteomic analyses to identify biomarkers of innate immunity and inflammation in chickens. Following albumin and IgG depletion from avian plasma, iTRAQ-based LC-MS/MS revealed 468 differentially abundant proteins (increased, 171; decreased, 297) in penguin sera, using an aspergillosis model system to investigate the proteomic, and inflammatory responses to *Aspergillus fumigatus* exposure [33]. It is notable that although 63 protein families were noted with increased abundance, and 17 individual proteins exhibited >4-fold abundance, while no significant detection of penguin complement proteins was evident. This suggests that complement does not play a role in the avian anti-Aspergillus response, as noted by [33]. Horvatić et al. used a tandem mass tag (TMT) approach linked to subsequent LC-MS/MS to quantitatively investigate the effect of LPS, and exposure time (up to 72 h), on specific protein abundance in chicken plasma (*n* = 12 chickens in total) [27]. Interestingly, these authors used plasma which had not previously been depleted of high abundance proteins prior to processing and LC-MS/MS. Overall, 87 significantly differentially abundant proteins were evident, of 418 total proteins detected, and identified including serum amyloid A (the major chicken acute phase protein), A1AGP and ovotransferrin in the LPS-treated cohort. Significantly elevated abundance of complement proteins was also observed by Horvatić et al. in the LPS-treated cohort of birds [27]. Using iTRAQ peptide labelling and LC-MS/MS, Shen et al. identified 239 differentially abundant proteins, when comparing laying and brooding hens (*n* = 6), out of a total of 1235 proteins detected in chicken serum following prior depletion of high abundance protein [34]. 

Although interest in, and use of, YCW-based products to improve agricultural outputs have grown enormously, to our knowledge no systematic and quantitative analysis of the serum proteome of supplemented animals has been investigated to gain an insight into the metabolic effects of YCW supplementation of animal feed. As far as could be found in the literature, no investigations using avian serum proteomics have been published on the effects of feed products on the broiler chicken. Here, we use the power of LC-MS/MS to reveal robust alterations in the broiler chicken serum proteome highly indicative of YCW supplement-associated immune priming.

## 2. Results

### 2.1. Overview of LC-MS/MS Results

Use of LC-MS/MS Compatible Reagent; (LCR) for protein re-solubilisation post-ProteoMiner^TM^ treatment was optimal and was used throughout this study (Supplementary Figures S1–S3). Consequently, tryptic digests, from 36 pooled serum samples from each control and GHP treated group, were analysed by LC-MS/MS at three time points, Day 7 (D7), Day 21 (D21) and Day 35 (D35). Within each time point, each treatment group was compared to control which created three comparator groups: GHP1 vs. control, GHP2 vs. control and GHP3 vs. control, for each time point. Within each comparator group, proteins which were significantly changed in abundance (*p* < 0.05) as well as proteins which were uniquely present in treatment groups in comparison to the control group or uniquely absent in treatment groups versus control, were identified. In addition, statistically significant alterations (*p* < 0.05) in protein abundance are also expressed as log2fold changes in Table 1, Table 2, Table 3, Table 4, Table 5, Table 6 and Table 7, whereby log2(1) = twofold or 100% alteration in abundance, etc.

Between 5% and 12% of the total identified proteins within each comparator group showed changes in abundance (Table 1). The largest change in protein abundance was seen on Day 7, with an average of 9.1% of proteins changed in abundance or unique to/absent from the serum of treated birds when compared to control birds. GHP1-treated birds showed the largest change in protein abundance throughout the three time points with an average of 7.8% of proteins changed in abundance. The largest individual change in abundance was seen in Day 7 GHP1 vs. control samples with 11.1% of proteins changed. 

**Figure 1:**
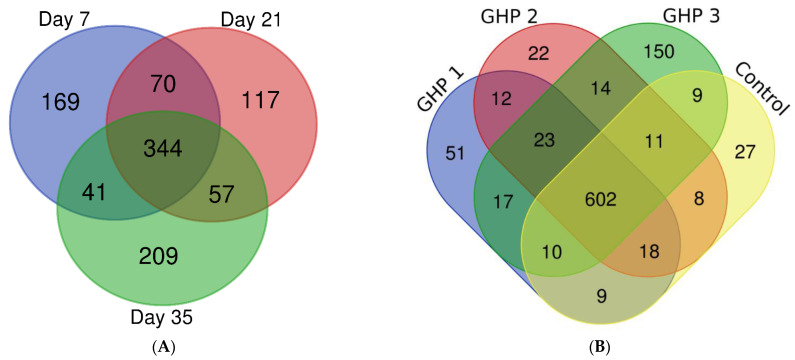
(**A**) Shared and distinct proteins among experimental time points. (**B**) Shared and distinct proteins among control and treatment groups. Figures were generated using the Venn Diagrams tool available at https://vandepeerlab.org/?q=tools/venn-diagrams (accessed on 15 February 2022).

Inter-day and inter-treatment analysis was also conducted on the total protein number identified throughout all serum samples analysed. This was achieved by applying LC-MS/MS data obtained from each sample to the data analysis software MaxQuant (Version 1.5.7.0; http://maxquant.org), first grouped by Day and then, in a separate analysis by treatment. Proteins (*n* = 1007) were identified in at least one pooled serum sample from all time points (Figure 1A). Over half of all proteins (*n* = 512) (51% of total proteins) were shared between at least two time points. Proteins (*n* = 169) were uniquely identified in pooled serum samples obtained on Day 7, on Day 21 (*n* = 117) and on Day 35 (*n* = 209). Shared proteins (*n* = 602) were identified in at least one serum sample from all treatment groups (Figure 1B). Most proteins (*n* = 733) (74% of total proteins) were shared between at least two treatment groups. Proteins (*n* = 27) were uniquely identified in pooled serum samples from the control treatment group, in the GHP2 treatment group (*n* = 22), in the GHP1 treatment group (*n* = 51) and the GHP3 treatment group (*n* = 150) (Figure 1B). 

### 2.2. Quantitative Proteomic Analysis of Serum from Broilers Fed a GHP1 Supplemented Diet vs. Serum from Broilers Fed on a Control Diet

**Figure 2:**
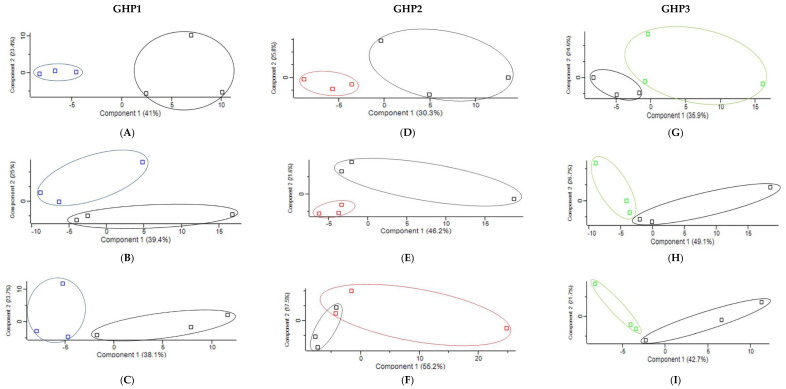
GHP1 Principal Component Analysis (PCA) comparing GHP1 (Blue) vs. day-specific control (Black) pooled serum samples for (**A**) Day 7, (**B**) Day 21, and (**C**) Day 35. GHP2 Principal Component Analysis (PCA) comparing GHP2 serum samples (Red) vs. day-specific control serum samples (Black) for (**D**) Day 7, (**E**) Day 21, and (**F**) Day 35. GHP3 Principal Component Analysis (PCA) comparing GHP3 (Green) vs. day-specific control (Black) pooled serum samples for (**G**) Day 7, (**H**) Day 21, and (**I**) Day 35.

**Figure 3:**
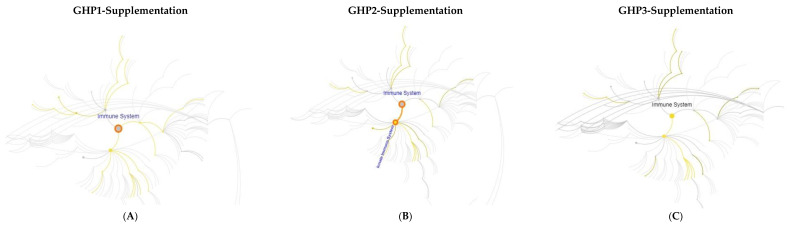
Reactome pathway map analysis of proteomic data following broiler dietary GHP supplementation over 35-day period. This data reveals consistent elevation of immune-function associated proteins indicative of an activated innate immune response associated with improved capacity to ameliorate infection. (**A**) Pathway map of 19 proteins involved in the immune system that were significantly increased in abundance or uniquely present in the serum of GHP1 supplemented broiler chickens. (**B**) Reactome pathway map of 19 proteins involved in the immune system that were significantly increased in abundance in the serum of GHP2 supplemented broiler chickens. Highlighted lines and dots represent proteomic pathways within the immune system that contain proteins which have been increased in abundance with GHP2 supplementation. (**C**) Pathway map of 19 proteins involved in the immune system that were significantly increased in abundance or uniquely present in the serum of GHP3 supplemented broiler chickens. All data obtained using Reactome.org [35].

Proteins (*n* = 84) were found to be significantly increased (*p* < 0.05) in abundance or uniquely present in the serum of GHP1 supplemented birds across the three time points (Table 2). Proteins (*n* = 62) were significantly decreased in abundance (*p* < 0.05) or not detected from GHP1 samples in comparison to control samples across the three time points (Table 3). Principal component analysis (PCA) (Figure 2A–C) of GHP1 serum samples versus control serum samples shows that GHP1 samples and control samples cluster separately on Day 7, Day 21 and Day 35. Hierarchal clustering was also conducted on proteomic data which matched PCA results on Day 7 and Day 21. However, one control sample on Day 35 clustered more closely with GHP1 samples than the control group. Using Reactome pathway mapping software, 19 proteins that were significantly increased (*p* < 0.05) in abundance or uniquely present in GHP1 samples were identified as involved in the innate immune system. These proteins are: Proteasome endopeptidase complex (A0A1L1RSU8), Proteasome subunit beta 1 (A0A1L1RYR5), proteasome 26S subunit, ATPase 5 (F1NU79), uncharacterized protein (F1NPN5), A1AGP (Q8JIG5), proteasome subunit alpha type (F1NC02), proteasome subunit alpha type (A0A1L1S0K9), proteasome subunit beta type (A0A1L1RUE7), proteasome subunit alpha type (A0A1D5PHL0), proteasome subunit alpha type (F1NEQ6), vascular cell adhesion molecule 1 (F1P201), elongation factor 1-alpha 1 (Q90835), proteasome subunit alpha type (Q5ZJX9), peroxiredoxin-6 (F1NBV0), complement Factor H (E1C7P4), interleukin 6 signal transducer (A0A1D5PMY8), glutaminyl-peptide cyclotransferase (A0A1D5PRR0), complement C4 precursor (A0A1D5P5V5), complement C5 (E1BRS7), complement C6 (B8ZX71) and complement C7 (E1C6U2). These proteins are represented in a Reactome pathway map (Figure 3A). Mannose binding protein (Q98TA4) was also increased in abundance and approaching significance (*p =* 0.16) on Day 35 in GHP1 samples (Supplementary Table S1). A few proteins involved in ROS detoxification were significantly increased (*p* < 0.05) in abundance on Day 7: specifically, peroxiredoxin 6 (F1NBV0), glutathione peroxidase (F1NPJ8) and glutaredoxin 3 (A0A1D5NW30) (Table 2). Additionally, proteins involved in vitamin A transport and metabolism were increased in abundance in GHP1 samples. Retinol binding protein 4 (P41263) level was significantly increased and retinol binding protein 7 (E1C0M1) was uniquely present in GHP1 samples on Day 7. The largest significant (*p* < 0.05) Log_2_ fold-change detected across all serum samples was observed in the Day 35 GHP1 vs. control comparator group. A1AGP (Q8JIG5) had a Log_2_ fold change of 2.12 on Day 35. Complementary and replicate ELISA analysis of pooled sera from Day 35 control versus GHP1-treated confirmed direction of elevated A1AGP levels (assay 1: 1.64 v 2.27 mg/mL and assay 2: 2.2 v 2.3 mg/mL) by previous LC-MS/MS approach, albeit not at statistical significance (Supplementary Figure S4). 

Several notable proteins showed changes in abundance in GHP1 samples. Osteocalcin (P02822) was uniquely detected in GHP1 samples on Day 21. Gastrokine-2 (A0A1D5PFM9) was uniquely present in GHP1 samples on Day 35. Transferrin receptor protein was significantly increased (*p* < 0.05) with a relatively large Log_2_ fold change (1.7) on Day 35. Insulin-like growth factor binding protein 5 (F1ND88) was significantly decreased (*p* < 0.05) on Day 35. Proteins (*n* = 5) with involvement in carbohydrate metabolism were altered in abundance (*p* < 0.05) with GHP1 supplementation. These include N-phosphoglycerate kinase (F1NU17), glyceraldehyde-3-phosphate dehydrogenase (P00356) and mimecan (Q9W6H0) which were significantly increased (*p* < 0.05) in abundance in GHP1 samples. N-acetyl-alpha-glucosaminidase (A0A1D5NU78) was uniquely present on Day 7. Beta-hexosaminidase (F1NTQ2) was increased in abundance and approaching significance (*p* = 0.09) on Day 7 (Supplementary Table S1). α-enolase (A0A1D5PSH6) was significantly decreased (*p* < 0.05) on Day 21. α-amylase was increased in abundance and approaching significance (*p* = 0.08) on Day 7 (Supplementary Table S1), also noted by McCaffrey et al. to be significantly increased in intestinal lumen of GHP supplemented broilers. β-enolase (P07322) showed decrease in abundance and approaching significance (*p* = 0.09) on Day 21 (Supplementary Table S1) and significantly decreased (*p* < 0.05) in abundance on Day 35 (Table 3) [8]. Proteins involved in nucleotide metabolism were significantly altered in abundance (*p* < 0.05) or unique to/absent from, GHP1 samples. Nucleoside diphosphate kinase (O57535) and cytidine/uridine monophosphate kinase 2 (R4GJC4) were uniquely present in GHP1 samples on Day 21 and Day 7, respectively. Adenylate kinase isoenzyme (P05081) was significantly increased (*p* < 0.05) on Day 21 and adenosine deaminase (Q5ZKP6) was significantly decreased (*p* < 0.05) on Day 21 and Day 35. 

### 2.3. Quantitative Proteomic Analysis of Serum from Broilers Fed a GHP2 Supplemented Diet vs. Serum from Broilers Fed a Control Diet

A total of eighty-two proteins were found to be significantly increased in abundance (*p* < 0.05) or uniquely present in sera from GHP2 fed birds compared to controls (Table 4). Proteins (*n* =55) were found to be significantly decreased (*p* < 0.05) in abundance or not detected in serum of GHP2 samples (Table 5). PCA of proteomic data from GHP2 and control sera (Figure 2D–F) showed that GHP2 samples and control samples cluster separately on Day 7, Day 21 and Day 35. Hierarchal clustering was also conducted on proteomic data which matched PCA results on Day 7, Day 21 and Day 35. Nineteen proteins, characterised to be involved in the innate immune system by Reactome pathway mapping software, were significantly increased (*p* < 0.05) in abundance in the GHP 2 serum samples: namely proteasome subunit beta 1 (A0A1L1RYR5), chromogranin A (F1NLZ2), proteasome 26S subunit, ATPase 5 (F1NU79), uncharacterized protein (Q5F491), surfactant protein A (Q90XB2), pantetheinase precursor (E1BUA6), uncharacterized protein (F1NPN5), alpha-1,4 glucan phosphorylase (E1BSN7), uncharacterized protein (A0A1D5PW77), T-complex protein 1 subunit theta (F1NEF2), proteasome subunit beta type (A0A1L1RUE7), transthyretin (P27731), elongation factor 1-alpha 1 (Q90835), proteasome subunit alpha type (F1NEQ6), metalloproteinase inhibitor 2 (R4GIL5), cathepsin D (Q05744), complement C4 Precursor (A0A1D5P5V5), complement C6 (B8ZX71) and complement C7 (E1C6U2). These are represented in a Reactome pathway map (Figure 3B). Mannose-binding protein (Q98TA4) was increased in abundance and approaching significance (*p* = 0.2) on Day 35 in GHP 2 samples.

Several proteins involved in carbohydrate metabolism were also significantly increased in abundance in GHP 2 samples. Specifically, glyceraldehyde-3-phosphate dehydrogenase (P00356) and phosphoglycerate kinase (F1NU17) were significantly increased (*p* < 0.05) in GHP 2 samples on Day 7. Individual proteins of note also showed changes in abundance in GHP 2 samples. Osteocalcin (P02822), a marker for bone resorption, was uniquely identified on Day 21. Gastrokine-2 (A0A1D5PFM9) was uniquely present in the serum of GHP 2 treated birds on Day 35. Insulin-like growth factor II was significantly decreased (*p* < 0.05) in abundance on Day 35 (Table 5). Transferrin receptor protein 1 (F1NTM6) was increased in abundance and approaching significance (*p* = 0.06) (Supplementary Table S2) on Day 35. Proteins (*n* = 3) involved in vitamin A transport [36,37] were increased in abundance in GHP 2 samples. Transthyretin (P27731) was significantly increased in abundance on Day 35, Retinol binding protein 7 (E1C0M1) was uniquely present on Day 7 and Retinol-binding protein 4 (P41263) was increased in abundance and approaching significance (*p* = 0.08) on Day 21 (Supplementary Table S2). Several proteins involved in nucleotide metabolism were significantly altered in abundance (*p* < 0.05) in GHP 2 samples. These were 3’-phosphoadenosine 5’-phosphosulfate synthase 1 (E1C8P2) which was uniquely present and adenylate kinase isoenzyme 1 (P05081) that was significantly increased (*p* < 0.05) on Day 7 (Table 4). Nucleoside diphosphate kinase (O57535) was uniquely present on Day 35 whereas adenosine deaminase (A0A1D5PDK4) was significantly decreased in abundance (*p* < 0.05) on Day 35 (Table 5). Finally, A1AGP was increased in abundance on Day 35 in samples from GHP 2 supplemented birds with a large fold change (Supplementary Table S3). However, this change was not significant (*p* = 0.37). 

### 2.4. Quantitative Proteomic Analysis of Serum from Broilers Fed a GHP3 Supplemented Diet vs. Serum from Broilers Fed on a Control Diet

Proteins (*n* = 64) were found to be significantly increased (*p* < 0.05) in abundance or uniquely present in GHP3 samples when compared to control, across all time points (Table 6). Proteins (*n* = 54) were significantly decreased (*p* < 0.05) or absent from GHP3 samples across all time points (Table 7). Figure 2G–I shows PCA of GHP3 serum samples versus control serum samples and reveals that GHP3 samples and control samples cluster separately on Day 7, Day 21 and Day 35. Hierarchal clustering was also conducted on proteomic data which did not match PCA grouping on Day 35, although samples largely grouped together on Day 7 and Day 21. Using the Reactome pathway mapping software [35], 19 proteins that were found to be significantly increased (*p* < 0.05) in abundance or uniquely present in GHP3 samples, were identified as involved in the innate immune response (Figure 3C). These proteins are: uncharacterised protein (F1NPN5), proteasome subunit beta 1 (A0A1L1RYR5), complement factor H (E1C7P4), proteasome 26S subunit, ATPase 5 (F1NU79), dual specificity phosphatase 3 (A0A1L1S0I4), chromogranin A (F1NLZ2), transthyretin (P27731), alpha-1,4 glucan phosphorylase (E1BSN7), proteasome subunit alpha type (F1NEQ6), β-2-microglobulin (P21611), β-hexosaminidase (F1NTQ2), complement C2 (A0A1D5P4P1), complement C4 precursor (A0A1D5P5V5), complement C5 (E1BRS7), complement C6 (B8ZX71), complement C7 (E1C6U2) and mannose-binding protein (Q98TA4) (Table 6). 

Proteins (*n* = 4) involved in carbohydrate metabolism showed changes in abundance in GHP3 samples. Glyceraldehyde-3-phosphate dehydrogenase (P00356) was significantly increased (*p* < 0.05) on Day 7 (Table 6). Glutamate dehydrogenase 1 and β-1,4-galactosyltransferase 4 (E1C9B0) were absent from GHP3 samples on Day 7 (Table 7). Proteins (*n* = 4) involved in nucleotide metabolism showed significant (*p* < 0.05) alterations in abundance in GHP3 samples. Adenylate kinase isoenzyme significantly increased (*p* < 0.05) on Day 7 (Table 6). Guanine deaminase (F1NJD6) significantly decreased (*p* <0.05) on Day 21. Adenosine deaminase (A0A1D5PDK4) significantly decreased (*p* < 0.05) on Day 35. Deoxythymidylate kinase (A0A1D5PKC2) was absent from GHP3 samples on Day 7 (Table 7).

Proteins (*n* = 2) involved in vitamin A transport increased in abundance in GHP3 samples. Retinol binding protein 4 (P41263) was increased in abundance and approaching significance (*p* = 0.06) on Day 21. Transthyretin (P27731) was significantly increased (*p* < 0.05) in abundance on Day 35 (Table 6). Many key proteins involved in the detoxification of ROS were significantly decreased (*p* < 0.05) in abundance or absent from GHP3 samples. Two catalases were absent from GHP3 samples on Day 7 and Day 21, respectively, catalase (A0A1D5PPU9) and catalase (Q5ZL24) (Table 7). Peroxiredoxin-1 (P0CB50) was significantly decreased in abundance on Day 35. Glutathione peroxidase (F1NPJ8) was increased in abundance and approaching significance (*p* = 0.08 and 0.09, respectively) in GHP3 samples on Day 21 and Day 35 (Supplementary Table S5). Glutathione S-transferase (GST) (Q08392) was absent from GHP3 samples in all time points, and this protein was identified as GST alpha-class. A few proteins showed changes in abundance in GHP3 samples whereby transferrin receptor protein was significantly increased (*p* < 0.05) on Day 35 and gastrin releasing peptide (A0A1D5PXC4) was significantly decreased (*p* < 0.05) in abundance on Day 35. 

GHP3-supplemented birds were supplemented an organic source of selenium in their diet instead of the inorganic source, selenium selenite, which was used for GHP1, GHP2 and control diets. To examine any potential effects of this change in selenium source, control and GHP3 samples were examined for a SeMet/SeCys substitution for methionine/cysteine using MAXQUANT [38]. Control and GHP3 samples were also searched for known selenoproteins. There was one protein identified to have SeMet/SeCys substitution in control samples (Supplementary Table S8A) which was SEC31 homolog B, COPII coat complex component (E1BXC8). The protein was detected in only one control sample with a sequence coverage of 3.6% and 1 peptide detected. Proteins (*n* = 4) were identified as having a SeMet/SeCys substitution in GHP3 samples (Supplementary Table S8B), coatomer subunit alpha (A0A1D5P185), golgin A4 (A0A1D5PNT3), nuclear factor related to kappaB binding protein (E1BZI6) and translocase of outer mitochondrial membrane 34 (F1P4X4). The sequence coverage of proteins identified was low, at 0.8–5.4%. Of the four proteins identified with a SeMet/SeCys substitution, three were identified in one GHP3 pooled serum sample obtained on Day 7. Using a list of previously identified selenoproteins obtained from Liu et al. [39] (Supplementary Table S7), one selenoprotein was identified in GHP3 and control samples, glutathione peroxidase (F1NPJ8) [39]. This protein was increased in abundance with *p*-value approaching significance on Day 21 (*p* = 0.08) and Day 35 (*p* = 0.09) (Supplementary Table S9). Selenoprotein F precursor (A0A1D5PFR6) was uniquely detected in GHP3 samples, but was not detected in GHP2, GHP1 and control samples. AIAGP was increased in abundance on Day 35 in samples from GHP3 supplemented birds with a large fold change (Supplementary Table S6), however, this was not significant (*p* = 0.36).

## 3. Discussion

### 3.1. Overview

Quantitative proteomic analysis has revealed that diet supplementation with GHP1, 2 and 3 significantly alters the abundance of specific serum proteins which contribute to the immune status and health of the broiler chicken. Consistent alterations in protein abundance were seen for those involved in the complement component of the innate immune system, nutrient transport, oxidative stress, and selenium status—all of which contribute to the overall animal health and provide evidential support for the supplemental effects of GHP products.

Supplementation of broiler diet with GHP products had a detectable effect on the avian serum proteome with averages of 6.31–7.78% of proteins significantly changed in abundance (*p* < 0.05) or unique/absent in the serum of GHP 1-, 2-and 3-, treated birds. GHP1 samples showed the largest number of protein changes with 85 proteins significantly increased in abundance (*p* < 0.05) or uniquely present in comparison to control, and 63 proteins significantly decreased in abundance (*p* < 0.05) or absent in comparison to control. GHP3 samples showed the lowest total number of proteins changed with 65 proteins significantly increased in abundance (*p* < 0.05) or uniquely present in comparison to control, and 55 proteins significantly decreased in abundance (*p* < 0.05) or absent in comparison to control. However, sera from GHP3 supplemented birds showed the greatest number of proteins that were unique to a treatment in inter-treatment analysis and, as will be discussed, GHP3 showed greater complement activation as well as alterations to serum seleno-protein abundance which may indicate a substantial impact on broiler health status.

### 3.2. Individual Proteins of Note

Several individual proteins of note were seen to be significantly altered in abundance (*p* < 0.05) or present/absent in treatment groups with interesting links to several aspects in animal health. Gastrokine-2 is secreted by gastric mucosal cells. This protein binds gastrokine-1 and is involved in regulating homeostasis of gastric mucosa [40]. It has been documented that GHP feed supplementation can have effects on gastric mucosa [41]. Gastokine-2 was uniquely present in the serum of GHP1 and GHP2 samples on Day 35. One other protein involved in the regulation of gastric mucosa is gastrin releasing peptide (A0A1D5PXC4). Abundance of this protein was significantly reduced (*p* < 0.05) in GHP3 samples on Day 35. Changes in the abundance of these proteins would indicate that these GHPs may have an effect on the regulation of the gastric mucosa.

Osteocalcin (P02822), uniquely present in GHP1 and GHP2 samples on Day 21, is a marker for bone turnover and its presence in serum is an indicator of bone resorption [36]. The presence of osteocalcin, only in the serum of birds supplemented with GHP1 and GHP2, could indicate that these products affect bone formation in the early stages of life which is of critical importance in chicken husbandry. Leg problems are prevalent in broilers [37] and can lead to higher culling rates in commercial production systems which have real monetary effects on the producer [38]. 

In GHP1 samples, GSH-Px was also seen to be increased in abundance on Day 7, which was also accompanied by a significant increase (*p* < 0.05) in two other enzymes involved in the detoxification of ROS: glutaredoxin 3 (A0A1D5NW30) and peroxiredoxin 6 (F1NBV0). This increase in the abundance of proteins involved in ROS detoxification may not be due to increased oxidative stress, but rather the stimulation of the production of these proteins by feed constituents. However, elucidating the molecular mechanisms of redox homeostasis maintenance in poultry and farm animals has received limited attention and are poorly characterized. Nevertheless, determining effective means of mitigating redox stress through nutritional modulation are exciting areas of research gaining momentum [42].

### 3.3. Immunological Effects

As noted, GHP products can have numerous immunological effects on animals [2,3,4,5,6,7]. Across all treatment groups, there were significant (*p* < 0.05) increases in the level of a number of complement components in the serum of GHP-supplemented broilers harvested on Day 35, which would indicate stimulation of the complement cascade by the GHP feed constituents. Complement C4 precursor (A0A1D5P4P1), Complement C6 (B8ZX71) and Complement C7 (E1C6U2) were significantly increased (*p* < 0.05) in all treatments samples on Day 35. Complement C5 (E1BRS7) was increased in abundance and approaching significance in GHP2 samples and was significantly increased (*p* < 0.05) in abundance in GHP1 and GHP3 samples. Complement C2 was significantly increased (*p* < 0.05) in abundance in GHP3 samples only. Indeed, GHP3 samples showed the greatest level of complement stimulation. Increases in serum levels of complement components indicate a stimulation of the complement cascade by these GHP feed supplements. 

A possible source of this complement stimulation by GHP products is through activation of the complement cascade by mannose binding protein (Q98TA4). This protein was significantly increased (*p* < 0.05) in abundance in GHP3 samples and increased and approaching significance in GHP2 (*p* = 0.2) and GHP1 (*p* = 0.162) samples on Day 35. Mannose binding protein, also called Mannose binding lectin (MBL) [43,44] is a C-type serum lectin [45] that binds mannose residues which results in C1-independent complement activation and binding leads to cleavage of C2 and C4 from their precursors by associated serine proteases [46]. Both C2 and C4 precursor were significantly increased in abundance (*p* < 0.05) in GHP3 samples. MBL may also interact directly with cell surface receptors which can initiate opsonophagocytosis [47] causing immunostimulation which could also explain the abundance rise in proteins which have been linked with the innate immune system. Interestingly, low serum concentrations of MBL has been linked to a greater susceptibility to infection [45]. There is little data available in the literature regarding stimulation of the chicken complement cascade by GHP feed supplementation. Slawinska et al. reported a downregulation of two genes influencing the complement system after *in ovo* administration of yeast-based prebiotics to broiler chickens followed by transcriptomic analysis [48]. Activation of the complement system by carbohydrate-based feed has been previously seen in aquaculture. Gilthead seabream were fed a diet supplemented with inulin, a branched carbohydrate comparable to β-glucan or MOS [49]. Supplementation resulted in significant increases (*p* < 0.05) in serum complement activity [50] which is in accordance with results found in the present study.

The increase in abundance of additional immunologically relevant proteins suggests immunostimulatory/immunomodulatory effects by these GHPs. One likely mediator of this immunostimulation is Surfactant Protein A (Q90XB2). This protein was uniquely present in GHP3 samples on Day 7, and is a C-type lectin receptor which is present in mucosal tissues and binds glycan ligands which can result in downstream immunological effects [51]. Carbohydrate recognition domains on this protein bind glycan residues resulting in innate immune stimulation [47] which could explain the increase in abundance of immune-related proteins observed. MOS-based products have been shown to elicit immunological responses without causing acute phase (fever) response [1]. Yet, the largest fold change was seen (Log_2_ Fold change = 2.12) for A1AGP in LC-MS/MS analyses. This protein is in high abundance in serum and has been previously characterised as an acute phase protein [26]. Large fold changes in A1AGP were also seen between samples from GHP2 and GHP3 supplemented birds and control samples (Log_2_ fold change = 3.69 and 1.86, respectively) although the *p*-value for these changes was not significant (*p* = 0.37 and 0.36, respectively). It is unlikely that the high fold changes noted for A1AGP indicate stimulation of the acute phase response because of the non-significant *p*-values (*p* < 0.05) seen in GHP2 and GHP3 as well as the lack of significant change (*p* < 0.05) in any other acute phase proteins (e.g., serum amyloid A). The data suggest that increases in the abundance of this protein may not represent an acute phase response but rather the natural variability of the serum level of this protein. Indeed, complementary A1AGP ELISA analysis of GHP1-treated and control samples revealed non-significant elevation in A1AGP in sera from GHP1-treated birds, in accordance with the results obtained from LC-MS/MS analysis.

### 3.4. Effects on Metabolism

With GHP supplementation, a number of proteins involved in carbohydrate and nucleotide metabolism were significantly altered (*p* < 0.05) in abundance across all treatment groups. Glyceraldehyde-3-phosphate dehydrogenase (P00356) was significantly increased (*p* < 0.05) in all treatment groups on Day 7. This protein is involved in glycolysis [52] and its increase could indicate a greater level of glycolysis in birds supplemented with these GHP products. Beta-hexosaminidase (F1NTQ2) was significantly increased (*p* < 0.05) in abundance in GHP3 samples and increased and approached significance (*p* = 0.08) in GHP1 samples on Day 7. This protein is involved in the release of N-acetylglucosamine and N-acetylgalactosamine from glycoproteins, which are in high concentration in the GHP [53] and its increase in abundance is likely a result of the increased glycoprotein substrate in the diet. Increase in the abundance of these proteins could indicate that the introduction of these feed supplements stimulated carbohydrate metabolism in broiler chickens. However, individual products had varying effects on the levels of carbohydrate metabolism proteins. GHP1 had a variable effect on carbohydrate metabolism. Proteins (*n =* 4), involved in carbohydrate metabolism were significantly increased in abundance (*p* < 0.05) or uniquely present in GHP1 samples. However, other proteins involved in carbohydrate metabolism showed a decrease in abundance such as α-Enolase (A0A1D5PSH6) and β-Enolase (P07322). GHP3 also had an inconsistent effect on the abundance of proteins involved in carbohydrate metabolism, with proteins both significantly increased (*p* < 0.05) and significantly decreased (*p* < 0.05) in abundance. Overall, the levels of proteins involved in carbohydrate metabolism were variable and, though significant changes (*p* < 0.05) were seen, no definitive effect on carbohydrate metabolism could be deduced.

Differential effects were also seen on proteins involved in nucleotide metabolism throughout all three products. Significant changes (*p* < 0.05) in the abundance of multiple proteins involved in this process would suggest an effect of GHP supplementation, but with proteins both increased and decreased in abundance, no common trend could be elucidated.

### 3.5. Vitamin A Transport

Vitamin A is of vital importance in the diet of the broiler chicken as it is not produced naturally by the animals and so must be obtained through the diet [54]. Deficiencies in broiler diets can lead to deterioration of reproductive [55] and immunological health [56,57]. Vitamin A is bound by Retinol binding protein 4 (RBP4) (P41263) which is in turn bound to Transthyretin (P27731) [58]. These proteins then transport Vitamin A throughout the animal. Retinol binding protein 7 (RBP7) (E1C0M1) is involved in the intracellular binding and transport of Vitamin A in cells [59]. RBP4 was significantly increased (*p* < 0.05) and RBP7 was uniquely identified in GHP1 samples on Day 7. Transthyretin was significantly increased on Day 35 in GHP2 samples and RBP7 was uniquely identified on Day 7, RBP4 was also increased in abundance and approached significance (*p* = 0.08) on Day 21. Transthyretin was significantly increased on Day 35 in GHP3 samples. Increases in the abundance of these proteins may indicate a greater abundance or availability of Vitamin A with the supplementation of these GHP products, however this requires further investigation. One common source of Vitamin A is β-carotene, a precursor to Vitamin A [60] that is naturally obtained through the consumption of carotenoid-producing organisms such as higher plants or photosynthetic microorganisms [61]. 

### 3.6. Effect of Organic Selenium Supplementation

Selenium is an essential element in the diet of broiler chickens and its supplementation has been seen to have beneficial effects on broiler health status and meat quality [62,63,64]. The dietary source of selenium can have an impact, as inorganic selenium is considered a pro-oxidant and organic selenium has been seen to have greater bioavailability [65,66,67]. Drip-loss has been linked to GSH-Px level [62]. GSH-Px was increased in abundance in all treatment groups with the largest fold-change noted in GHP3 samples where it was increased and approaching significance on Day 21 and Day 35. GSH-Px level has been previously shown to be influenced by selenium supplementation and a greater bioavailability of organic selenium from GHP3 would explain this increased GSH-Px level. A reduction in drip-loss and increase in serum GSH-Px has been reported previously in birds supplemented with organic selenium rather than its inorganic form [68,69]. This result was also matched in pigs with significantly higher (*p* < 0.01) levels of serum GSH-Px in organic selenium supplemented pigs at 0.2mg/kg and 0.3 mg/kg supplementation [70]. In this work, selenium was also supplemented to broiler diets at 0.3 mg/kg (inorganic selenium selenite for GHP1 and GHP2, Se yeast for GHP3). 

The absence of the potentially pro-oxidative inorganic selenium in the diet of GHP3 supplemented birds can also be seen in the significant reduction or absence of proteins involved in the detoxification of ROS: Catalase (*n* = 2), A0A1D5PPU9 and Q5ZL24, which were also absent from GHP3 samples on Day 7 and Day 21, respectively. The protective effects of selenium have been seen in organs such as the liver and organic forms of selenium have been liked to reduced liver damage [66]. Glutathione *S*-transferase α (αGST) (Q08392) is an enzyme involved in the detoxification of reactive xenobiotics and is present in high concentrations in the cytosol of hepatocytes [71,72,73]. This enzyme conjugates glutathione to xenobiotics [74] and its presence in serum has previously been characterised as an indicative biomarker for acute hepatitis or liver damage [75]. αGST is absent from the GHP3 samples on Day 7, Day 21 and Day 35 which would indicate that this product may confer some hepatoprotective effect on the broiler chicken. This absence could be due to the reduced level of oxidative damage with an organic selenium source, the greater bioavailability of organic selenium leading to increased levels of GSH-Px which reduces oxidative damage or a dual effect of these two factors. Evidence suggests that organic selenium has a greater bioavailability than its inorganic forms [65] and this hepatoprotective effect could also be a result of the greater bioavailability of organic selenium allowing greater protective potential of enzymes that make use of the element. A greater bioavailability of organic selenium from GHP3 was not only supported by the increased levels of GSH-Px on all days, but also by the higher number of proteins detected with SeMet/SeCys substitutions and the unique presence of selenoprotein F precursor in the serum of GHP3 treated birds. An increased bioavailability might not only lead to increased benefits to the broiler chicken, but also to the producer and consumer. 

## 4. Materials and Methods 

### 4.1. Experimental Design, Sample Collection and Preservation 

**Figure 4:**
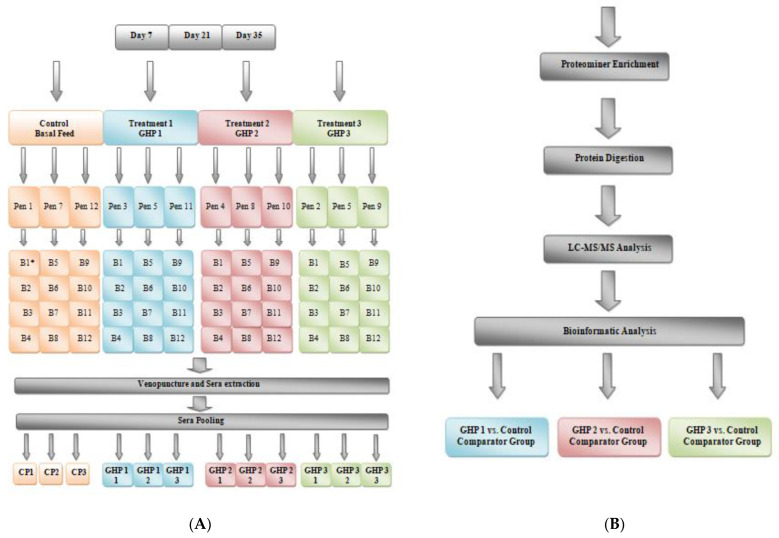
(**A**) Overview of serum sample collection, preparation, and analysis from sample collection to serum pooling. (**B**) Serum analysis from Proteominer^TM^ enrichment to Bioinformatic Analysis. * B1 denotes Bird 1.

A total of 492 day-of-hatch male broiler chickens were used in the poultry feeding trial. Clean concrete-floor pens were used to house the birds in a medium scale trial facility on-site at Agri-Food Biosciences Institute (AFBI) (Belfast, UK). Animals were randomly split into four groups of 3 pens, with 12 pens in total (41 birds/pen; 123 birds/group) using a randomized complete block design (Figure 4A). The pens were divided into four groups: group 1, fed a basal diet; group 2–4, fed a basal diet which included supplements GHP1, 2 and 3, respectively, at 1 kg/tonne in starter, grower and finisher diets. These supplements were mannan-rich fractions extracted from the yeast cell wall of *Saccharomyces cerevisiae*. Additional individual GHP details and the manufacturers recommended inclusion levels are published elsewhere (Alltech Inc., Lexington, KY, USA) [30]. Basal diets were prepared by a commercial feed mill and consisted primarily of wheat and soybean meal which met full dietary nutritional requirements [76]. Starter diets were fed from day 0 to day 10, grower diets from day 11–25 and finisher diets, day 26 to day 35. Feed and water were provided *ad libitum* throughout the study. Each pen was dressed in fresh litter for bedding from day zero. The temperature was initially set at 30 °C per day up to day 10 and then decreased linearly by 1 °C every second day. Birds received a lighting regimen of 16 h light and 8 h darkness until day 35. All conditions were kept uniform for all four groups. On days 7, 21 and 35, blood samples from necropsied birds were collected into 100 mL sterile sample cups (75.562.105; Sarstedt) and then transferred to BD Vacutainer^®^ blood collection tubes using 10 mL wide-bore serological pipettes (86.1688.010; Sarstedt). The whole blood was allowed to clot at room temperature for 30–60 min. The clot was removed by centrifugation at 4500 rpm for 10 min at 4 °C. The resulting serum supernatant was apportioned into 0.5 mL aliquots and snap frozen using liquid nitrogen. Aliquots were stored at −80 °C and thawed on ice before use. 

All procedures were subject to the approval of the local Animal Welfare Ethics Review Board and subsequent approval by a Home Office Inspector. All procedures were carried out under the strict guidelines of the Animal (Scientific Procedures) Act 1986 [76].

### 4.2. ProteoMiner™ Serum Enrichment Evaluation

Individual and pooled avian sera were first cleared of precipitate by centrifugation (10,000× *g*, 10 min). Sera (200 μL) were then applied to the ProteoMiner™ Protein Enrichment kit (#1633006; BioRad) according to manufacturer’s instructions. After loading and washing, 20 μL of either the provided ProteoMiner elution buffer or 0.1 M Tris-HCL, 6 M Urea and 2 M Thiourea pH 8.0 (LC-MS/MS Compatible Reagent; LCR) was applied to the column. Columns were then lightly vortexed for 5 s over 15 min. Caps were removed and columns placed in capped collection tubes and centrifuged (1000× *g*, 1 min). This elution step was repeated twice giving 60 μL of enriched serum sample. Enriched serum samples were stored −20 °C until LC-MS/MS analysis (Supplementary Figures S1–S3) which showed LCR gave improved protein detection. 

### 4.3. Upgraded ProteoMiner™ Processing for Serum Enrichment

All pooled serum samples were first cleared of precipitate by centrifugation (10,000× *g*, 10 min). Sera (200 μL) were then applied to the ProteoMiner™ Protein Enrichment kit according to manufacturer’s instructions. After washing, deionised water (200 μL) was applied to columns which were rotated end-over-end for 1 min and drained through centrifugation (1000× *g*, 1 min). 20 μL of LCR was applied to the column. Columns were then lightly vortexed for 5 s over 15 min. Caps were removed and columns placed in capped collection tubes and centrifuged (1000× *g*, 1 min). This elution step was repeated twice giving 60 μL of enriched serum sample. Enriched serum samples were stored at −20 °C until further processing.

### 4.4. Protein Digestion and Q-Exactive Liquid Chromatography-Mass Spectrometry (LC-MS/MS) Analysis

Enriched serum samples were removed from −20 °C and allowed to reach room temperature. Any precipitate was resuspended through light vortexing. Samples were tested for protein concentration by Bradford assay [77]. 10 μL of the enriched sample was then separated. Remaining samples were returned to −20 °C storage. The pH of the sample was then adjusted to between 8.5 and 9.0 using 3 to 5 volumes of Label-Free Solubilisation Buffer. The volume of diluted enriched serum sample corresponding to 5 μg protein was then removed for further processing. Protein samples (5 μg each) were then brought to 10 mM Dithiothreitol (DTT) using 0.05 M DTT and incubated at 56 °C for 30 min. Samples were then brought to 25 mM Iodoacetamide (IAA) using 0.11 M IAA. Lys-C (0.5 μL) was then added to samples at 1:100 ratio (Protease: Protein) and samples were incubated at 37 °C for 4 h. 50 mM ammonium bicarbonate (3 volumes) was then added to samples. Trypsin (0.8 μL) was then added to samples at 1:25 ratio (Protease: Protein). Protease Max (0.45 μL) was then added. Samples were incubated at 37 °C overnight. Using Sample Buffer (0.33 volumes), samples were then diluted. Peptide samples were evaporated to dryness in a SpeedVac™ (DNA 120; Thermo Scientific, Waltham, MA, USA) and stored at −20 °C prior to ZipTip treatment and transfer to Q Exactive mass spectrometer vials for injection (Figure 4B). Peptide samples were then analysed using a Thermo Scientific™ Q Exactive connected to a Dionex Ultimate™ 2000 (RSLCnano) chromatography system. Each sample was loaded onto an EASY-Spray™ PepMap RSLC C18 column (75 μm × 500 mm) and separated by an increasing acetonitrile gradient over 120 min at a flow rate of 250 nL/min [78,79]. The mass spectrometer was operated in positive mode with MS^n^ carried out on the 15 most abundant precursor ions at each time point. Singly charged ions were excluded from analysis.

### 4.5. Label-Free Quantitative (LFQ) Proteomic Analysis Using MaxQuant and Perseus

Peptide-Spectral mapping and protein peptide matching of raw files from Q-Exactive analysis of LFQ proteomic analysis were carried out using MaxQuant (version 1.5.7.0; http://maxquant.org), accessed on 9 April 2021 [80]. This utilised the Andromeda database search to match MS/MS data with the *Gallus gallus* 9031 reference proteome (UP000000539–*Gallus gallus*) from UniProt (http://uniprot.org, accessed on 9 April 2021). Search parameters included: peptide tolerance (ppm) of 20 for first search and 4.5 for main search, carbidomethylation of cysteine as a fixed modification, oxidation of methionine and acetylation of N-termini as variable modifications, maximum 2 missed cleavage sites, and a minimum 1 peptide detected per protein. The maximum protein/peptide false discovery rates were set at 1% based on a comparison to a reverse database (decoy database). The LFQ algorithm was used to generate normalised spectra intensities to infer relative protein abundance. Subsequently, protein groups were exported and processed in Perseus (version 1.5.6.0; http://coxdocs.org/doku.php?id=:perseus:start; accessed on 15 April 2021) for data filtering [81]. Proteins (i) only identified by site (ii) only identified by modification site, or (iii) identified by the decoy database were removed. Extracted LFQ intensities measured for each run were grouped according to treatment and time point. Protein abundances were log_2_ transformed.

### 4.6. A1AGP Immunoassay

A chicken A1AGP enzyme-linked immunosorbent assay (ELISA) (ab157690) (abcam^®^) was used for complementary analysis of serum A1AGP levels accordingly to the manufacturer’s instructions.

### 4.7. Software and Statistical Analysis

Overall, 36 pooled serum samples were prepared, extracted, and analysed by LFQ proteomics. These comprised of Control pool (Day-specific) and 3 Test pools at day 7, 21 and 35 (4 × 3 × 3 = 36). Thus, at each time point (Day 7, Day 21, and Day 35), treatment groups were compared to each day-specific control group creating three comparator groups (GHP1 vs. Control, GHP2 vs. Control and GHP3 vs. Control). A 2-sample t-test was performed to identify proteins with significant differences (*p* < 0.05) within each comparator group. Proteins uniquely detected in a treatment group and not detected in control, or proteins not detected in treatment and detected in control groups, were identified and tabulated. Pathway mapping was carried out using Reactome Pathway Database (https://reactome.org; accessed on 30 April 2021). Proteins were specifically analysed for features and/or functions in UniProt (http://uniprot.org).

All statistical analyses were carried out using GraphPad Prism (version 5.0; GraphPad Software Inc.) or Microsoft Excel. 

## 5. Conclusions

The significant alterations in proteins involved in several key processes would suggest that these products had effects on the systemic health status of the broiler chicken that were measurable through serum quantitative proteomic analysis. In this first study of its type, the multifaceted effects of GHP supplementation including stimulation of the innate immune system, particularly the complement system, alterations in the levels of a few proteins involved in the detoxification of ROS, stimulation of metabolic processes, gastric mucosal development and transport of iron and Vitamin A, were noted. The three YCW-based products showed similarity regarding their effects, which would be expected from three related products. We recognise that the use of sample pooling may influence the precise nature of the observed changes in protein abundance. However, individual results also show that these three products have product-specific effects which may be elucidated with further study. Future investigations should ideally deploy the use of smaller pools or individual serum samples to detect alterations in discrete protein abundance.

Future animal challenge studies, using bacterial (e.g., *Escherichia coli* or *Campylobacter jejuni*) or fungal pathogens (e.g., *Aspergillus fumigatus*) will ultimately be required to definitively elucidate the protective effects of the GHP diets studied herein.

## Figures and Tables

**Table 1:** Overview of proteins with significantly changed abundance within comparator groups.

Comparator Group	Total Identified Proteins	Significant changes *	Unique/Absent Changes **	Total Changes	Total Changes (%)
D7 GHP1 vs. D7 control	651	51	21	72	11.06
D7 GHP2 vs. D7 control		38	26	64	9.83
D7 GHP3 vs. D7 control		14	28	42	6.45
D21 GHP1 vs. D21 control	588	34	9	43	7.31
D21 GHP2 vs. D21 control		25	9	34	5.78
D21 GHP3 vs. D21 control		23	10	33	5.61
D35 GHP1 vs. D35 control	624	21	10	31	4.97
D35 GHP2 vs. D35 control		21	17	38	6.09
D35 GHP3 vs. D35 control		29	14	43	6.89

* Significant changes denote proteins which have changed in abundance with *p* < 0.05 in a two-sample *t*-test. In addition, statistically significant alterations (*p* < 0.05) in protein abundance are also expressed as log2fold changes in Table 2, Table 3, Table 4, Table 5, Table 6 and Table 7, whereby log2(1) = twofold or 100% alteration in abundance, etc. ** Unique/Absent Changes denote proteins that were uniquely present in a treatment group or absent from a treatment group when compared to day-specific control.

**Table 2:** Proteins with significantly (*p* < 0.05) **increased** abundance or unique in GHP1 vs. control serum samples. Proteins are listed in order of change of abundance.

Protein Description	Fold Change ^1^	Peptides	Coverage (%) ^2^	Day ^3^	Accession
N-acetyl-alpha-glucosaminidase	Unique	2	3.7	Day 7	A0A1D5NU78
Uncharacterized protein	Unique	2	1	Day 7	A0A1D5NZ61
Dynactin subunit 2	Unique	6	22.1	Day 7	A0A1D5PGQ9
Uncharacterized protein	Unique	5	41.4	Day 7	A0A1D5PQ15
Proteasome endopeptidase complex	Unique	3	43.7	Day 7	A0A1L1RSU8
Proteasome subunit beta 1	Unique	3	36.4	Day 7	A0A1L1RYR5
Retinol binding protein 7	Unique	3	32.1	Day 7	E1C0M1
Uncharacterized protein	Unique	4	30.8	Day 7	F1N8Y3
Elongation factor 1-alpha	Unique	15	49.9	Day 7	F1N9H4
Succinyl-CoA:3-ketoacid-coenzyme A transferase	Unique	3	11	Day 7	F1N9Z7
Endoplasmic reticulum lectin 1	Unique	3	12	Day 7	F1NCV8
Small nuclear ribonucleoprotein 13	Unique	3	30.5	Day 7	F1NII6
PDZ and LIM domain 5	Unique	4	8.2	Day 7	F1NTC8
Proteasome 26S subunit, ATPase 5	Unique	3	12.7	Day 7	F1NU79
Natriuretic peptides A (Prepronatriodilatin)	Unique	3	17.9	Day 7	P18908
Profilin	Unique	2	27.9	Day 7	Q5ZL50
Twisted gastrulation protein homolog 1	Unique	2	15.6	Day 7	Q98T89
Cytidine/uridine monophosphate kinase 2	Unique	6	26.5	Day 7	R4GJC4
Nucleoside diphosphate kinase	Unique	2	20.3	Day 21	O57535
Osteocalcin	Unique	3	45.4	Day 21	P02822
Tenascin	Unique	8	8.7	Day 21	P10039
Gastrokine 2	Unique	2	18.6	Day 35	A0A1D5PFM9
N-acetylglucosamine-1-phosphate transferase γ subunit	Unique	2	16	Day 35	E1BS68
N-acetylglucosamine-1-phosphate transferase γ subunit	0.34	5	27.2	Day 7	E1BS68
Adhesion G protein-coupled receptor G6	Unique	2	2.5	Day 35	E1C8C2
Alpha-1-anti-ase	Unique	9	27.8	Day 35	F1NPN5
Aggrecan core protein	Unique	3	2.3	Day 35	F1NZX1
Alpha-1-acid glycoprotein	2.12	7	13.9	Day 35	Q8JIG5
Transferrin receptor protein 1	1.7	10	10.5	Day 35	Q90997
Heat shock protein beta-1	1.3	18	77.9	Day 7	F1P593
Myosin light chain 1, skeletal muscle isoform	1.23	9	54.9	Day 7	P02604
Myosin regulatory light chain 2, skeletal muscle isoform	1.22	9	57.7	Day 7	P02609
Adenylate kinase isoenzyme 1	1.15	11	58.1	Day 7	P05081
Glyceraldehyde-3-phosphate dehydrogenase	1.13	20	72.1	Day 7	P00356
Proline and arginine rich end leucine rich repeat protein	1.13	10	27.9	Day 7	A0A1D5PAN0
Glycerol-3-phosphate dehydrogenase	1.11	9	24	Day 7	A0A1D5P1Y7
Proteasome subunit alpha type	1.08	7	36.2	Day 7	F1NC02
Uncharacterized protein	1.08	7	21.1	Day 7	F1NIP5
N-Phosphoglycerate kinase	0.94	17	51.9	Day 7	F1NU17
Complement C5	0.93	82	54.8	Day 35	E1BRS7
Dynactin subunit 3	0.89	6	32.9	Day 7	A0A1D6UPU1
Glutaredoxin 3	0.88	8	29.8	Day 7	A0A1D5NW30
Uncharacterized protein	0.86	7	15.2	Day 7	A0A1L1RQM3
Low mol. weight phosphotyrosine protein phosphatase	0.84	10	58.5	Day 7	A0A1D5P9Z1
Proteasome subunit alpha type	0.84	9	46.3	Day 7	A0A1L1S0K9
Protein/nucleic acid deglycase DJ-1	0.84	5	51.4	Day 7	A0A1D5PN39
Complement C6	0.83	29	33.4	Day 35	B8ZX71
Proteasome subunit beta type	0.79	13	56.6	Day 7	A0A1L1RUE7
Proteasome subunit alpha type	0.79	5	36.1	Day 7	A0A1D5PHL0
Periostin	0.77	16	24.5	Day 21	F1P4N9
Receptor of-activated protein C kinase 1	0.76	9	40.9	Day 7	A0A1I7Q3Y2
Proteasome subunit alpha type	0.76	6	23.9	Day 7	F1NEQ6
Serpin H1	0.76	12	34.9	Day 7	P13731
Vascular cell adhesion molecule 1	0.75	6	13	Day 21	F1P201
Ribosomal protein S14	0.69	4	34.9	Day 7	Q5ZHW8
T-complex 1	0.68	9	17.6	Day 7	Q5ZMG9
Uncharacterized protein	0.67	8	38.3	Day 7	A0A1D5PAH2
Complement C7	0.66	28	52.3	Day 35	E1C6U2
T-complex protein 1 subunit zeta	0.64	20	51.8	Day 7	Q5ZJ54
Elongation factor 1-alpha 1	0.63	18	54	Day 7	Q90835
Retinol-binding protein 4	0.61	11	68.9	Day 7	P41263
Proteasome subunit alpha type	0.6	6	41.5	Day 7	Q5ZJX9
Nuclear transport factor 2	0.55	3	33.9	Day 7	F1NLL4
Chaperonin containing TCP1 subunit 5	0.55	17	34.4	Day 7	Q5F411
40S ribosomal protein S12	0.54	7	61.4	Day 7	P84175
Uncharacterized protein	0.54	6	39.8	Day 35	A0A1D5PK48
Peroxiredoxin-6	0.53	12	66.4	Day 7	F1NBV0
Uncharacterized protein	0.51	6	40.2	Day 35	A0A1D5PZU8
Eukaryotic translation initiation factor 5A-1	0.5	6	56.6	Day 7	Q09121
Glia maturation factor beta	0.5	5	31.6	Day 7	A0A1D6UPR3
Complement C4 precursor	0.48	61	45.6	Day 35	A0A1D5P5V5
Collagen type V alpha 1 chain	0.46	6	4.8	Day 21	F1NI79
Insulin-like growth factor binding protein acid labile subunit	0.44	8	15.4	Day 21	F1NI07
TRK-fused gene	0.41	4	12.9	Day 7	A0A1L1RK44
Complement factor B-like protease	0.39	6	36.8	Day 7	P81475
Nidogen 2	0.36	20	16.3	Day 35	F1NDL4
Uncharacterized protein	0.35	5	17.6	Day 7	Q5ZMC1
Complement factor H	0.32	93	71.5	Day 7	E1C7P4
Glutathione peroxidase	0.3	11	45.9	Day 7	F1NPJ8
Interleukin 6 signal transducer	0.29	3	5	Day 7	A0A1D5PMY8
Sortilin	0.28	14	21.3	Day 7	A0A1D5PNT8
Glutaminyl-peptide cyclotransferase	0.26	13	58.5	Day 7	A0A1D5PRR0
Fibromodulin	0.23	2	3.7	Day 21	P51887
Mimecan	0.15	9	32.7	Day 35	Q9W6H0

^1^ Fold change refers to the log_2_ fold change in protein abundance in response to GHP1 treatment; Unique refers to proteins only identified in GHP1 serum compared to control serum. ^2^ Coverage (%) refers to the % of protein sequence represented by identified peptides. ^3^ Day refers to the time point at which the differently abundant protein was detected: Day 7, Day 21 or Day 35 of feed trial. Proteins referred to in text are highlighted in (red).

**Table 3:** Proteins with significantly (*p* < 0.05) **decreased** abundance or absent in GHP1 vs. control serum samples. Proteins are listed in order of change of abundance.

Protein Description	Fold Change ^1^	Peptides	Coverage (%) ^2^	Day ^3^	Accession
Malate dehydrogenase	Absent	3	23.3	Day 7	A0A1D5PZS3
Junction plakoglobin	Absent	6	10.4	Day 7	E1C1V3
Uncharacterized protein	Absent	2	19.1	Day 7	R4GIC2
DEAD-box helicase 17	Absent	3	6.1	Day 21	A0A1D5PD32
Uncharacterized protein	Absent	3	9	Day 21	A0A1D5PF52
V-type proton ATPase subunit B, brain isoform	Absent	3	11.3	Day 21	A0A1D5PP57
Splicing factor proline and glutamine rich	Absent	3	18.4	Day 21	A0A1D5PPW4
Glutathione S-transferase	Absent	2	9.5	Day 21	Q08392
T-complex protein 1 subunit theta	Absent	3	6.8	Day 21	Q6EE31
NSF attachment protein alpha	Absent	2	8.2	Day 35	A0A1D5NUZ0
Uncharacterized protein	Absent	3	2.5	Day 35	A0A1D5NW21
Glia maturation factor beta	Absent	2	28	Day 35	A0A1D6UPR3
Uncharacterized protein	Absent	5	11.7	Day 35	E1BSP1
Heterogeneous nuclear ribonucleoprotein A2/B1	Absent	5	20.1	Day 35	Q5ZME1
Serpin family F member 2	−2.17	13	24.2	Day 7	F1NAR5
Beta-enolase	−1.65	12	40.9	Day 35	P07322
Receptor of-activated protein C kinase 1	−1.33	6	21.9	Day 21	A0A1I7Q3Y2
Serpin family G member 1	−1.24	12	15.8	Day 7	F1NA58
Uncharacterized protein	−1.18	2	0.7	Day 7	A0A1L1RLW1
Tubulin beta-7 chain	−1.18	12	40.1	Day 21	P09244
Uncharacterized protein	−1.11	11	8.6	Day 21	A0A1L1RJ91
Phosphoglycerate kinase	−1.03	11	27.8	Day 21	A0A1D5NZW9
Fibulin 5	−0.85	7	17.2	Day 35	A0A1L1RQ98
Elongation factor 1-alpha 1	−0.84	16	49.7	Day 21	Q90835
Pyruvate kinase PKM	−0.83	26	45.6	Day 35	P00548
Adenosine deaminase	−0.8	17	60.4	Day 35	Q5ZKP6
DAZ associated protein 1	−0.79	4	15.4	Day 21	Q5ZM92
Alpha-enolase	−0.74	14	34.3	Day 21	A0A1D5PSH6
Chaperonin containing TCP1 subunit 5	−0.73	9	22.25	Day 21	Q5F411
Macrophage receptor with collagenous structure	−0.71	10	21.4	Day 21	A0A1D5PJZ3
Adenosine deaminase	−0.7	20	69.8	Day 21	A0A1D5PDK4
Tubulin alpha chain	−0.68	11	30.3	Day 21	A0A1D5PC38
Uncharacterized protein	−0.68	10	30.3	Day 21	E1C477
Heterogeneous nuclear ribonucleoprotein K	−0.68	3	8.4	Day 21	A0A1L1S010
T-complex protein 1 subunit zeta	−0.67	14	31.2	Day 21	Q5ZJ54
Phosphoglycerate mutase 1	−0.62	9	25.4	Day 35	Q5ZLN1
Tubulin beta-6 chain	−0.62	14	38.2	Day 21	P09207
LSM8 homolog, U6 small nuclear RNA associated	−0.6	4	55.9	Day 21	E1BZ75
Chaperonin containing TCP1 subunit 2	−0.57	11	26.5	Day 21	Q5F424
Alpha-actinin-1	−0.56	4	4.8	Day 21	A0A1D5P9P3
Uncharacterized protein	−0.54	7	18.5	Day 21	F1NIP5
Complement C1q C chain	−0.52	5	24	Day 21	A0A1D5PGB2
Kininogen 1	−0.52	15	28.1	Day 7	A0A1L1RNR4
Complement C1q B chain	−0.51	6	33.6	Day 21	F1NH19
Insulin-like growth factor binding protein 5	−0.5	3	19.3	Day 35	F1ND88
Vitronectin	−0.48	15	39.5	Day 7	E1C7A7
Fibulin-1	−0.46	23	39.3	Day 35	O73775
Ubiquilin 4	−0.46	5	12.4	Day 21	A0A1D5P624
Methylthioribose-1-phosphate isomerase 1	−0.46	9	46.6	Day 21	A0A1D5PN97
EGF containing fibulin like extracellular matrix protein 1	−0.45	19	52.7	Day 35	A0A1D5P380
Far upstream element binding protein 1	−0.45	12	21.5	Day 21	A0A1D5P2H3
Apolipoprotein H	−0.44	14	42.7	Day 35	A0A1L1RTQ4
Epiphycan	−0.43	5	13.7	Day 7	Q90944
Ig lambda chain C region	−0.42	9	53.3	Day 35	P20763
Aggrecan core protein	−0.38	16	7.7	Day 7	F1NZX1
Fibulin-1	−0.38	18	31.8	Day 21	A0A1L1RU28
Uncharacterized protein	−0.34	43	25.4	Day 35	F1NEQ4
Far upstream element-binding protein 2	−0.3	13	18.7	Day 21	Q8UVD9
F-actin-capping protein subunit beta isoforms 1 and 2	−0.29	11	36.2	Day 21	P14315
Uncharacterized protein	−0.28	2	38.5	Day 21	F1NSC8
Vimentin	−0.27	30	64.6	Day 21	A0A1L1RXL9
Fibrinogen gamma chain	−0.18	9	33.7	Day 7	E1BV78

^1^ Fold change refers to the log_2_ fold change in protein abundance in response to GHP1 treatment. ^2^ Coverage (%) refers to the % of protein sequence represented by identified peptides. ^3^ Day refers to the time point at which the differently abundant protein was detected: Day 7, Day 21 or Day 35 of feed trial. Proteins referred to in text are highlighted in (red).

**Table 4:** Proteins with significantly (*p* < 0.05) **increased** abundance or unique in GHP2 vs. control serum samples. Proteins are listed in order of change of abundance.

Protein Description	Fold change ^1^	Peptides	Coverage (%) ^2^	Day ^3^	Accession
Barrier to autointegration factor 1	Unique	2	42.2	Day 7	A0A1D5NXY4
Uncharacterized protein	Unique	2	1.0	Day 7	A0A1D5NZ61
Dynactin subunit 2	Unique	6	22.1	Day 7	A0A1D5PGQ9
Legumain	Unique	2	11.8	Day 7	A0A1L1RX51
Proteasome subunit beta 1	Unique	3	36.4	Day 7	A0A1L1RYR5
Retinol binding protein 7	Unique	3	32.1	Day 7	E1C0M1
Ankyrin repeat domain 2	Unique	2	8.9	Day 7	E1C1Q6
Mediator of cell motility 1	Unique	3	21.9	Day 7	E1C6C0
3’-phosphoadenosine 5’-phosphosulfate synthase 1	Unique	5	12.3	Day 7	E1C8P2
Uncharacterized protein	Unique	4	30.8	Day 7	F1N8Y3
Elongation factor 1-alpha	Unique	15	49.9	Day 7	F1N9H4
Succinyl-CoA:3-ketoacid-coenzyme A transferase	Unique	3	11.0	Day 7	F1N9Z7
Four and a half LIM domains 1	Unique	4	18.5	Day 7	F1NED9
Myeloid protein 1	Unique	6	30.1	Day 7	F1NEF7
mall nuclear ribonucleoprotein 13	Unique	3	30.5	Day 7	F1NII6
Chromogranin A	Unique	2	10.7	Day 7	F1NLZ2
PDZ and LIM domain 5	Unique	4	8.2	Day 7	F1NTC8
Proteasome 26S subunit, ATPase 5	Unique	3	12.7	Day 7	F1NU79
Cofilin-2	Unique	3	25.9	Day 7	P21566
Uncharacterized protein	Unique	2	3.7	Day 7	Q5F491
Profilin	Unique	2	27.9	Day 7	Q5ZL50
Endophilin-A2	Unique	4	20.4	Day 7	Q8AXV0
Surfactant protein A	Unique	3	16.2	Day 7	Q90XB2
Seryl-tRNA synthetase	Unique	2	4.3	Day 7	R4GJ59
Disintegrin and metalloproteinase domain-containing protein 33 precursor	Unique	2	3.6	Day 21	A0A1D5NV10
Calpastatin	Unique	2	3.9	Day 21	A0A1D5PFJ2
Tubulointerstitial nephritis antigen like 1	Unique	2	10	Day 21	F1N8G6
Osteocalcin	Unique	3	45.4	Day 21	P02822
Tenascin	Unique	8	8.7	Day 21	P10039
Tenascin	Unique	10	10.8	Day 35	P10039
Gastrokine-2	Unique	2	18.6	Day 35	A0A1D5PFM9
N-acetylglucosamine-1-phosphate transferase γ subunit	Unique	2	16	Day 35	E1BS68
Pantetheinase precursor	Unique	10	27	Day 35	E1BUA6
Adhesion G protein-coupled receptor G6	Unique	2	2.5	Day 35	E1C8C2
Alpha-1-anti-ase	Unique	9	27.8	Day 35	F1NPN5
Nucleoside diphosphate kinase	Unique	3	28.1	Day 35	O57535
Apolipoprotein B (Fragment)	Unique	7	24.7	Day 35	P11682
Glycerol-3-phosphate dehydrogenase	1.55	9	37.5	Day 7	A0A1D5P1Y7
Adenylate kinase isoenzyme 1	1.44	11	63.4	Day 7	P05081
Glyceraldehyde-3-phosphate dehydrogenase	1.33	20	75.1	Day 7	P00356
Myosin regulatory light chain 2, skeletal muscle isoform	1.22	9	64.3	Day 7	P02609
Heat shock protein beta-1	1.15	18	79.4	Day 7	F1P593
Myosin light chain 1, skeletal muscle isoform	1.08	9	60.9	Day 7	P02604
Phosphoglycerate kinase	1.07	17	60.9	Day 7	F1NU17
Proline and arginine rich end leucine rich repeat protein	1	10	32.9	Day 7	A0A1D5PAN0
Uncharacterized protein	0.97	7	25.5	Day 7	F1NIP5
Protein/nucleic acid deglycase DJ-1	0.91	5	59	Day 7	A0A1D5PN39
Actin, alpha skeletal muscle	0.89	13	24.4	Day 21	A0A1I7Q414
Uncharacterized protein	0.84	2	3.7	Day 21	F1NMN2
Carbonic anhydrase 2	0.83	7	44.6	Day 35	P07630
Alpha-1,4 glucan phosphorylase	0.81	5	7.8	Day 35	E1BSN7
Uncharacterized protein	0.8	54	24	Day 21	A0A1D5PW77
Receptor of-activated protein C kinase 1	0.75	9	56.9	Day 7	A0A1I7Q3Y2
Complement C7	0.73	28	54.3	Day 35	E1C6U2
Low mol. weight phosphotyrosine protein phosphatase	0.69	10	60.8	Day 7	A0A1D5P9Z1
Uncharacterized protein	0.69	7	24.1	Day 7	A0A1L1RQM3
T-complex protein 1 subunit theta	0.65	8	21	Day 7	F1NEF2
Proteasome subunit beta type	0.65	14	25.1	Day 21	A0A1L1RUE7
Transthyretin	0.64	9	72.7	Day 35	P27731
Cystatin A	0.62	4	18.3	Day 21	F1NHG8
Tropomyosin alpha-1 chain	0.61	16	57.2	Day 7	A0A1D5NVL7
Serpin H1	0.58	12	44	Day 7	P13731
60 kDa heat shock protein	0.57	12	34.4	Day 7	Q5ZL72
Complement C6	0.56	29	40.3	Day 35	B8ZX71
Elongation factor 1-alpha 1	0.53	18	58.9	Day 7	Q90835
Ribosomal protein L23a	0.53	3	24.5	Day 7	E1BS06
Proteasome subunit alpha type	0.53	15	27.9	Day 21	F1NEQ6
Collagen type V alpha 1 chain	0.49	11	57	Day 21	F1NI79
Eukaryotic translation initiation factor 5A-1	0.47	6	63.9	Day 7	Q09121
C-type lectin domain family 3-member B	0.45	6	40.5	Day 21	Q9DDD4
Metalloproteinase inhibitor 2	0.42	11	67.7	Day 21	R4GIL5
F-actin-capping protein	0.38	6	6.7	Day 21	P14315
Uncharacterized protein	0.37	5	41.1	Day 21	A0A1L1S0T3
Cathepsin D	0.37	5	87.6	Day 21	Q05744
Sortilin	0.35	13	67.7	Day 21	A0A1D5PNT8
Sortilin	0.28	14	25.6	Day 7	A0A1D5PNT8
Uncharacterized protein	0.33	5	52.9	Day 7	A0A1D5PH37
Complement C4 precursor	0.3	61	59.2	Day 35	A0A1D5P5V5
Heparan sulfate proteoglycan 2	0.24	8	42.6	Day 21	A0A1L1RJ69
Fibromodulin (FM)	0.14	24	62.7	Day 21	P51887
Far upstream element-binding protein 2	0.13	13	44.1	Day 21	Q8UVD9

^1^ Fold change refers to the log_2_ fold change in protein abundance in response to GHP2 treatment; Unique refers to proteins only identified in GHP2 serum compared to control serum. ^2^ Coverage (%) refers to the % of protein sequence represented by identified peptides. ^3^ Day refers to the time point at which the differently abundant protein was detected: Day 7, Day 21 or Day 35 of feed trial. Proteins referred to in text are highlighted in (red).

**Table 5:** Proteins with significantly (*p* < 0.05) **decreased** abundance or absent in GHP2 vs. control serum samples. Proteins are listed in order of change of abundance.

Protein Description	Fold Change ^1^	Peptides	Coverage (%) ^2^	Day ^3^	Accession
Growth differentiation factor 11	Absent	2	6.4	Day 7	A0A1D5P7V6
Uncharacterized protein	Absent	5	10.3	Day 7	F1NZV7
Uncharacterized protein	Absent	3	9	Day 21	A0A1D5PF52
Glutathione S-transferase	Absent	2	9.5	Day 21	Q08392
Catalase	Absent	2	8.9	Day 21	Q5ZL24
T-complex protein 1 subunit theta	Absent	3	6.8	Day 21	Q6EE31
NSF attachment protein alpha	Absent	2	8.2	Day 35	A0A1D5NUZ0
Uncharacterized protein	Absent	3	2.5	Day 35	A0A1D5NW21
Fibulin-1	Absent	6	36.5	Day 35	A0A1L1RU28
ERH, mRNA splicing and mitosis factor	Absent	17	39.4	Day 35	A0A1L1RZP8
Endoplasmic reticulum lectin 1	Absent	2	8.5	Day 35	F1NCV8
Nuclear transport factor 2	Absent	2	63	Day 35	F1NLL4
Nuclear transport factor 2	−0.6	4	40.7	Day 21	F1NLL4
TAR DNA-binding protein 43 (TDP-43)	Absent	4	14.5	Day 35	Q5ZLN5
Heterogeneous nuclear ribonucleoprotein A2/B1	Absent	3	20.1	Day 35	Q5ZME1
Junction plakoglobin	−1.45	33	7.1	Day 7	E1C1V3
Uncharacterized protein	−1.23	12	0.7	Day 7	A0A1L1RLW1
Insulin like growth factor binding protein 5	−1.17	3	15.9	Day 35	F1ND88
Fibromodulin	−1.08	4	6.3	Day 35	P51887
Fibulin 5	−1.02	7	16.7	Day 35	A0A1L1RQ98
Serpin family D member 1	−1.00	21	37.9	Day 21	A0A1D5PLZ2
Uncharacterized protein	−0.93	2	37.5	Day 7	F1NSC7
Uncharacterized protein	−0.33	3	37.5	Day 21	F1NSC7
Olfactomedin-like protein 3	−0.92	20	50.5	Day 35	Q25C36
Ovoinhibitor	−0.82	9	60.6	Day 7	P10184
EGF containing fibulin like extracellular matrix protein 1	−0.78	19	53.7	Day 35	A0A1D5P380
Hemoglobin subunit alpha-D	−0.77	7	90.1	Day 7	P02001
Fibrinogen beta chain	−0.65	8	26.9	Day 7	Q02020
Carboxypeptidase	−0.62	7	16	Day 21	A0A1L1RXB2
Fibrinogen gamma chain	−0.61	6	34.1	Day 7	E1BV78
Insulin-like growth factor II	−0.59	2	11	Day 35	P33717
Uncharacterized protein	−0.59	21	23.5	Day 7	A0A1D5PSJ4
Histone H2B 8	−0.58	11	19	Day 7	Q9PSW9
Uncharacterized protein	−0.53	3	36.4	Day 21	F1NC22
Chemerin	−0.52	7	43.3	Day 35	A0A0K0PUH6
Uncharacterized protein	−0.52	2	38.5	Day 21	F1NSC8
Matrilin-3	−0.5	53	27.9	Day 7	O42401
Matrilin-3	−0.38	7	16.8	Day 35	O42401
Uncharacterized protein	−0.48	25	52	Day 7	R9PXM5
Hyaluronan binding protein 2	−0.48	15	31.4	Day 21	F1NEB3
Uncharacterized protein	−0.47	8	58.7	Day 21	A0A1D5PV72
Ig lambda chain C region	−0.45	9	50.7	Day 21	P20763
Protein-lysine 6-oxidase	−0.43	7	28	Day 35	A0A1D5P1U0
Adenosine deaminase	−0.4	17	66	Day 35	A0A1D5PDK4
HGF activator	−0.37	11	15.6	Day 7	E1BZN8
Collagen alpha 1(VI) chain	−0.34	10	12.7	Day 35	A0A1D5PWN6
Apolipoprotein A-I	−0.25	2	90.3	Day 7	P08250
Transforming growth factor beta induced	−0.25	8	70.3	Day 7	A0A1D5NX81
CD74 molecule	−0.21	4	20.8	Day 35	F1NYL5
ADAM metallopeptidase with thrombospondin type 1 motif 13	−0.12	29	27.1	Day 35	A0A1D5PEF7

^1^ Fold change refers to the log_2_ fold change in protein abundance in response to GHP2 treatment. ^2^ Coverage (%) refers to the % of protein sequence represented by identified peptides. ^3^ Day refers to the time point at which the differently abundant protein was detected: Day 7, Day 21 or Day 35 of feed trial. Proteins referred to in text are highlighted in (red).

**Table 6:** Proteins with significantly (*p* < 0.05) **increased** abundance or unique in GHP3 vs. control serum samples. Proteins are listed in order of change of abundance.

Protein Description	Fold Change ^1^	Peptides	Coverage (%) ^2^	Day ^3^	Accession
Barrier to autointegration factor 1	Unique	2	42.2	Day 7	A0A1D5NXY4
HSPA (Hsp70) binding protein 1	Unique	5	12.1	Day 7	A0A1D5P628
Uncharacterized protein	Unique	9	41.4	Day 7	A0A1D5PQ15
Proteasome subunit beta 1	Unique	4	36.4	Day 7	A0A1L1RYR5
Ankyrin repeat domain 2	Unique	5	8.9	Day 7	E1C1Q6
Plexin domain containing 2	Unique	3	5.9	Day 7	E1C486
Mediator of cell motility 1	Unique	2	21.9	Day 7	E1C6C0
3’-phosphoadenosine 5’-phosphosulfate synthase 1	Unique	3	12.3	Day 7	E1C8P2
Elongation factor 1-alpha	Unique	3	49.9	Day 7	F1N9H4
Four and a half LIM domains 1	Unique	3	18.5	Day 7	F1NED9
Small nuclear ribonucleoprotein 13	Unique	4	30.5	Day 7	F1NII6
Chromogranin A	Unique	3	10.7	Day 7	F1NLZ2
PDZ and LIM domain 5	Unique	3	8.2	Day 7	F1NTC8
Proteasome 26S subunit, ATPase 5	Unique	2	12.7	Day 7	F1NU79
Uncharacterized protein	Unique	2	14.1	Day 7	F1NWB2
Natriuretic peptides A	Unique	2	17.9	Day 7	P18908
Cofilin-2	Unique	4	25.9	Day 7	P21566
Stratifin	Unique	15	28.5	Day 7	R4GF89
Seryl-tRNA synthetase	Unique	3	4.3	Day 7	R4GJ59
Disintegrin and metalloproteinase domain-containing protein 33 precursor	Unique	2	3.6	Day 21	A0A1D5NV10
Gastrokine 2	Unique	3	18.6	Day 35	A0A1D5PFM9
Dual specificity phosphatase 3	Unique	2	26.3	Day 35	A0A1L1S0I4
Alpha-1-anti-ase	Unique	2	27.8	Day 35	F1NPN5
Aggrecan core protein	Unique	2	2.3	Day 35	F1NZX1
Insulin-like growth factor I	Unique	3	19.0	Day 35	P18254
Zyxin	Unique	5	4.8	Day 35	Q04584
Adenylate kinase isoenzyme 1	1.12	11	60.3	Day 7	P05081
Transferrin receptor protein 1	1.07	10	9.7	Day 35	F1NTM6
Myosin regulatory light chain 2, skeletal muscle isoform	1.07	9	57.8	Day 7	P02609
Uncharacterized protein	1.04	2	19.2	Day 35	A0A1D5P1L5
Complement C5	1.04	82	55.5	Day 35	E1BRS7
Heat shock protein beta-1	1.03	18	77.9	Day 7	F1P593
Transthyretin	1.00	9	71.4	Day 35	P27731
Glyceraldehyde-3-phosphate dehydrogenase	0.95	20	72.0	Day 7	P00356
Uncharacterized protein	0.94	4	37.9	Day 21	A0A1L1RQF3
Myosin light chain 1, skeletal muscle isoform	0.90	9	53.9	Day 7	P02604
Alpha-1,4 glucan phosphorylase	0.89	5	6.5	Day 35	E1BSN7
Uncharacterized protein	0.79	6	39.8	Day 35	A0A1D5PK48
Actin, alpha skeletal muscle	0.78	13	34.8	Day 21	A0A1I7Q414
Low mol. weight phosphotyrosine protein phosphatase	0.75	10	58.5	Day 7	A0A1D5P9Z1
Uncharacterized protein	0.70	7	21.5	Day 7	F1NIP5
Complement C6	0.68	29	34.6	Day 35	B8ZX71
Complement C7	0.68	28	52.0	Day 35	E1C6U2
Uncharacterized protein	0.67	4	37.1	Day 35	A0A1L1RQF3
Uncharacterized protein	0.63	18	22.3	Day 35	A0A1L1S0T3
Uncharacterized protein	0.60	2	23.0	Day 21	A0A1D5P058
Uncharacterized protein	0.56	5	42.3	Day 35	F1NSC7
Complement C4 precursor	0.55	61	48.1	Day 35	A0A1D5P5V5
Mannose-binding protein	0.50	8	27.5	Day 35	Q98TA4
Proteasome subunit alpha type	0.45	6	23.5	Day 7	F1NEQ6
Beta-2-microglobulin	0.45	5	60.5	Day 21	P21611
Uncharacterized protein	0.44	4	38.5	Day 35	F1NSC8
Beta-hexosaminidase	0.42	20	49.2	Day 7	F1NTQ2
Sortilin	0.41	14	20.9	Day 7	A0A1D5PNT8
RAD23 homolog B, nucleotide excision repair protein	0.41	28	9.0	Day 7	F1N9B7
Complement C2	0.41	2	15.7	Day 35	A0A1D5P4P1
Vascular cell adhesion molecule 1	0.39	9	12.3	Day 21	F1P201
Uncharacterized protein	0.38	6	21.3	Day 21	A0A1L1S0T3
Complement factor H	0.28	15	71.9	Day 21	E1C7P4
Plasminogen	0.23	94	69.7	Day 21	R4GMH5
Apolipoprotein A-I	0.20	57	92.8	Day 21	P08250
Uncharacterized protein	0.19	55	17.3	Day 7	Q5ZMC1
Lumican	0.17	5	39.2	Day 35	P51890
Prolyl 4-hydroxylase subunit alpha-1	0.13	11	21.5	Day 7	P16924

^1^ Fold change refers to the log_2_ fold change in protein abundance in response to GHP3 treatment; Unique refers to proteins only identified in GHP3 serum compared to control serum.^2^ Coverage (%) refers to the % of protein sequence represented by identified peptides. ^3^ Day refers to the time point at which the differently abundant protein was detected: Day 7, Day 21 or Day 35 of feed trial. Proteins referred to in text are highlighted in (red).

**Table 7:** Proteins with significant (*p* < 0.05) **decreased** abundance or absent in GHP3 vs. control serum samples. Proteins are listed in order of change of abundance.

Protein Description	Fold Change ^1^	Peptides	Coverage (%) ^2^	Day ^3^	Accession
Glutamate dehydrogenase 1, mitochondrial	Absent	4	9.9	Day 7	A0A1D5NT61
Growth differentiation factor 11	Absent	2	6.4	Day 7	A0A1D5P7V6
Catalase	Absent	3	5.3	Day 7	A0A1D5PPU9
Malate dehydrogenase	Absent	3	23.3	Day 7	A0A1D5PZS3
Integral membrane protein 2B	Absent	2	46.2	Day 7	A0A1L1RIU5
Uncharacterized protein	Absent	2	0.9	Day 7	A0A1L1RLW1
Beta-1,4-galactosyltransferase 4	Absent	2	8.5	Day 7	E1C9B0
Carbamoyl-phosphate synthase 1	Absent	4	3.3	Day 7	F1N9N8
Glutathione S-transferase	Absent	2	9.5	Day 7	Q08392
NSF attachment protein alpha	Absent	2	8.2	Day 21	A0A1D5NUZ0
Uncharacterized protein	Absent	3	9	Day 21	A0A1D5PF52
Deoxythymidylate kinase	Absent	3	22.6	Day 21	A0A1D5PKC2
Lysophospholipase II	Absent	2	19	Day 21	E1BRI5
Small nuclear ribonucleoprotein 13	Absent	3	36.7	Day 21	F1NII6
Proteasome 26S subunit, ATPase 5	Absent	3	12.7	Day 21	F1NU79
Glutathione S-transferase	Absent	2	9.5	Day 21	Q08392
Catalase	Absent	2	8.9	Day 21	Q5ZL24
Carbohydrate sulfotransferase 3	Absent	4	13.3	Day 21	Q92179
NSF attachment protein alpha	Absent	2	8.2	Day 35	A0A1D5NUZ0
Uncharacterized protein	Absent	3	2.5	Day 35	A0A1D5NW21
Reversion inducing cysteine rich protein with kazal motifs	Absent	6	8.9	Day 35	A0A1D5PTW4
Uncharacterized protein	Absent	3	29.6	Day 35	A0A1D5PZ95
Glutathione S-transferase	Absent	2	9.5	Day 35	Q08392
TAR DNA-binding protein 43	Absent	3	14.5	Day 35	Q5ZLN5
Heterogeneous nuclear ribonucleoprotein A2/B1	Absent	5	20.1	Day 35	Q5ZME1
Cytidine/uridine monophosphate kinase 2	Absent	5	25.7	Day 35	R4GJC4
Gastrin-releasing peptide	−1.30	3	19.7	Day 35	A0A1D5PXC4
Fibromodulin	−1.20	4	7.7	Day 35	P51887
Tubulin beta-7 chain	−1.20	12	39.7	Day 21	P09244
Beta-tropomyosin	−1.18	27	62.2	Day 35	Q05705
Integrin-linked kinase	−1.11	4	10.3	Day 35	Q9DF58
cAMP-dependent protein kinase type I-alpha regulatory subunit	−1.02	11	27.4	Day 35	Q5ZM91
Fibulin 5	−0.90	7	16.8	Day 35	A0A1L1RQ98
cAMP-dependent protein kinase type I-alpha regulatory subunit	−0.89	6	20.3	Day 21	Q5ZM91
Tropomyosin alpha-1 chain	−0.84	12	47.6	Day 21	A0A1D5P342
MHC class II beta chain 2	−0.82	3	14.8	Day 21	A5HUL4
Elongation factor 1-alpha 1	−0.77	16	50.0	Day 21	Q90835
Beta-tropomyosin	−0.71	22	61.3	Day 21	Q05705
Hyaluronan binding protein 2	−0.70	15	32.5	Day 21	F1NEB3
Phosphatidylcholine-sterol acyltransferase	−0.68	13	37.4	Day 21	P53760
Heat shock protein beta-1	−0.67	17	79.4	Day 35	F1P593
Collagen alpha-2(I) chain	−0.65	11	10.1	Day 35	P02467
Complement C7	−0.61	21	43.8	Day 7	E1C6U2
Alpha-actinin-1	−0.48	4	4.37	Day 21	A0A1D5P9P3
DAZ associated protein 1	−0.47	4	14.0	Day 21	Q5ZM92
Adenosine deaminase	−0.41	17	65.6	Day 35	A0A1D5PDK4
Alpha-actinin-1	−0.41	15	12.7	Day 35	A0A1D5P9P3
Chemerin	−0.41	7	46.2	Day 35	A0A0K0PUH6
Collagen type XVIII alpha 1 chain	−0.40	5	4.7	Day 35	A0A1D5P5M7
Guanine deaminase	−0.38	8	23.5	Day 21	F1NJD6
Macrophage receptor with collagenous structure	−0.33	10	21.4	Day 21	A0A1D5PJZ3
Peroxiredoxin-1	−0.30	8	45.5	Day 35	P0CB50
Fibulin-1	−0.16	18	31.1	Day 21	A0A1L1RU28
Heterogeneous nuclear ribonucleoprotein M	−0.15	6	7.7	Day 21	F7B5K7

^1^ Fold change refers to the log_2_ fold change in protein abundance in response to GHP3 treatment. ^2^ Coverage (%) refers to the % of protein sequence represented by identified peptides. ^3^ Day refers to the time point at which the differently abundant protein was detected: Day 7, Day 21 or Day 35 of feed trial. Proteins referred to in text are highlighted in (red).

## Data Availability

All data in both manuscript and Supplementary data files.

## Supplementary Materials

The supporting information can be downloaded at: https://www.mdpi.com/article/10.3390/ijms231911844/s1.
