# Supplementary material for: Quantitative Proteomic Analysis Reveals Yeast Cell Wall Products Influence the Serum Proteome Composition of Broiler Chickens
Source: Int J Mol Sci. 2022 Oct 6;23(19):11844. doi: 10.3390/ijms231911844 (PMC9569515; doi:10.3390/ijms231911844)
Supplement: Supplementary file 1 [file ijms-23-11844-s001.zip › ijms-1910575-supplementary.pdf]

Quantitative Proteomic Analysis Reveals Yeast Cell Wall Products Influence the Serum Proteome Composition of Broiler Chickens

Niall Conlon [1,2], Richard A. Murphy [2], Aoife Corrigan [2], Sean Doyle [1], Rebecca A. Owens [1] and Sheena Fagan [2,*]

Results

S1. Preliminary proteomic analysis of broiler serum

Twelve serum samples were used in preliminary proteomic analysis. These serum samples were from individual broiler chickens harvested on Day 35, three serum samples from each treatment group. Serum samples were enriched for low abundance proteins using Proteominer™ enrichment technology. Enriched serum samples were then analysed through LC-MS/MS. **Figure S1A** shows a frequency distribution graph of the number of proteins and sequence coverage of proteins detected across the serum samples (n = 12) prepared using the commercial Proteominer™ buffer. A total of 380 proteins were detected across samples applied to the LC-MS/MS in preliminary analysis. Of detected proteins, 35% were detected with less than 10% sequence coverage. **Figure S1B** shows a total ion chromatogram of a serum sample prepared using the commercial Proteominer™ elution buffer. Detection levels are low throughout this LC-MS/MS analytical run until 125 min, when a large peak can be seen. The m/z for this peak is 615.4. This m/z corresponds to CHAPS, a component of the commercial Proteominer™ elution buffer. This large CHAPS peak (125 min-130 min) (Blue Arrow) caused suppression in peptide detection within the enriched serum sample. The base peak of this chromatogram is 1.39E10.

S2. Development and analysis of LC-MS/MS Compatible Reagent (LCR)

To avoid the signal suppression effect seen in proteomic samples prepared using the commercial Proteominer™ elution buffer, an alternative reagent was developed, lacking CHAPS but capable of eluting proteins from the enrichment column. This buffer, LCR, was used in place of the commercial Proteominer™ elution buffer during protein enrichment. One serum sample was enriched for LAP using the Proteominer™ technology with LCR used in place of the commercial elution buffer. The enriched serum sample was then analysed by LC-MS/MS. **Figure S2A** shows a total ion chromatogram of the serum sample prepared using LCR. This total ion chromatogram lacks the large peak between 125 min and 130 min that was previously seen in samples prepared using the commercial

Proteominer™ elution buffer (**Figure S1B**). Higher levels of peptide detection can be seen throughout this LC-MS/MS analysis when compared to **Figure S1B**. The base peak for the total ion chromatogram (**Figure S2A**) is 1.30E10.

A.

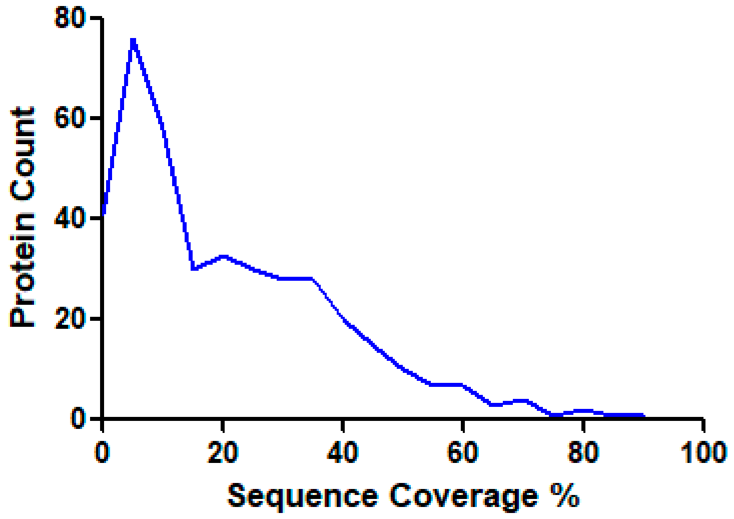

B.

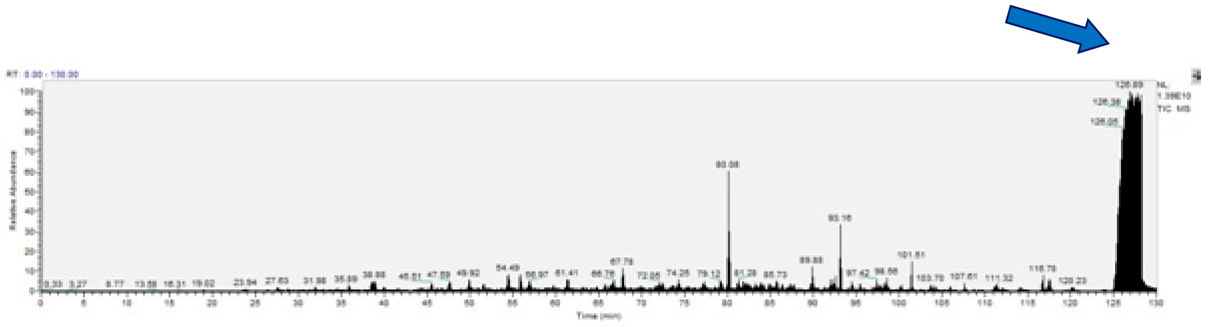

Supplementary Figure S1. A. Frequency distribution graph of samples prepared using the commercial Proteominer™ elution buffer showing number of proteins identified and protein sequence coverage. **B.** Total Ion Chromatograph of one representative sample prepared using the Proteominer™ elution buffer. Arrow indicates CHAPS elution.

The number of proteins detected, and sequence coverage of detected proteins were compared between data obtained from analyses done on a serum sample prepared using the commercial Proteominer™ elution buffer and the same serum sample prepared using LCR. It can be seen in **Figure S2B** that a greater number of proteins were detected, and higher sequence coverage was achieved using LCR whereby 218 proteins were detected in the serum sample prepared using the commercial Proteominer™ buffer, but 475 proteins were detected in the serum sample prepared using LCR.

Proteins (n =218) were identified in the serum sample prepared using the commercial Proteominer™ buffer (**Figure S2B**). However, 475 proteins were detected in the serum sample prepared

using LCR. Of these proteins, 196 were identified by both analyses. Proteins (*n* = 22) were uniquely identified in the sample prepared using the Proteominer™ elution buffer and 279 proteins (over 10-fold more) were uniquely identified in the sample prepared using LCR.

A.

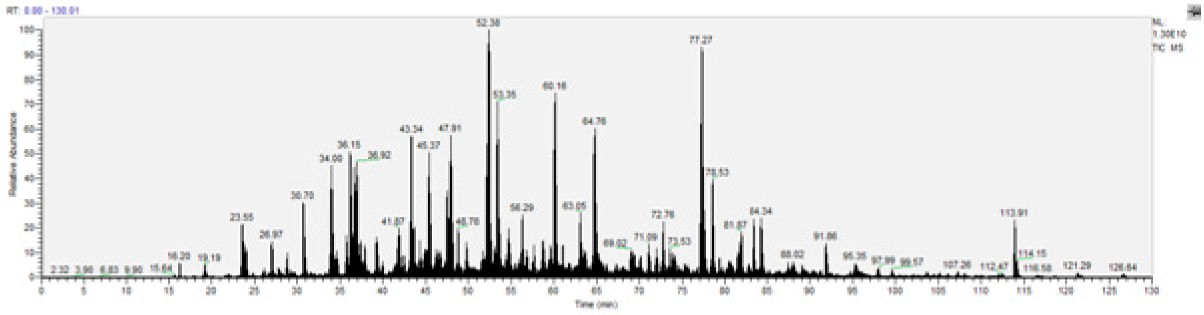

B.

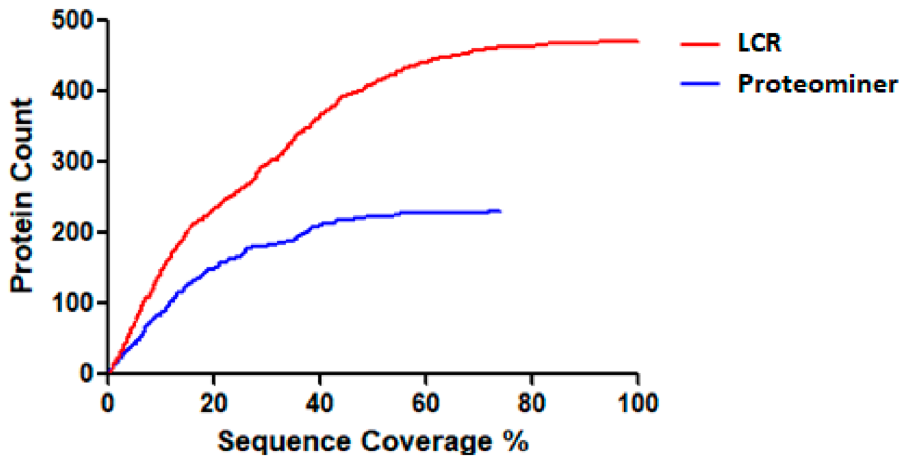

Supplementary Figure S2. A. Total Ion Chromatograph of serum sample prepared using LCR. **B.** Cumulative frequency distribution graph comparing proteins detected in samples prepared using the commercial Proteominer™ elution buffer and LCR.

S3. Comparison of sample groups prepared using Proteominer Elution Buffer and LCR

To further evaluate the effects of the replacement elution buffer on larger sample groups, twelve serum samples prepared using the Proteominer elution buffer were compared to twelve serum samples prepared using LCR. Samples prepared using the commercial Proteominer™ buffer are from three individual birds obtained from each Gut Health Product (GHP) group on Day 35. Samples prepared using LCR are serum samples pooled by pen on Day 35. All serum samples were enriched for low abundance proteins using the Proteominer™ small-capacity enrichment kit and analysed through LC-MS/MS. It can be seen in **Figure S3A, B** that a greater number of proteins were detected, and higher sequence coverage was achieved using the LCR reagent. There was a 55% increase in the total number of proteins identified and an 88% increase in the number of proteins identified with greater than 10% sequence coverage in samples prepared using LCR, when compared with samples prepared using the commercial Proteominer™ elution buffer.

A

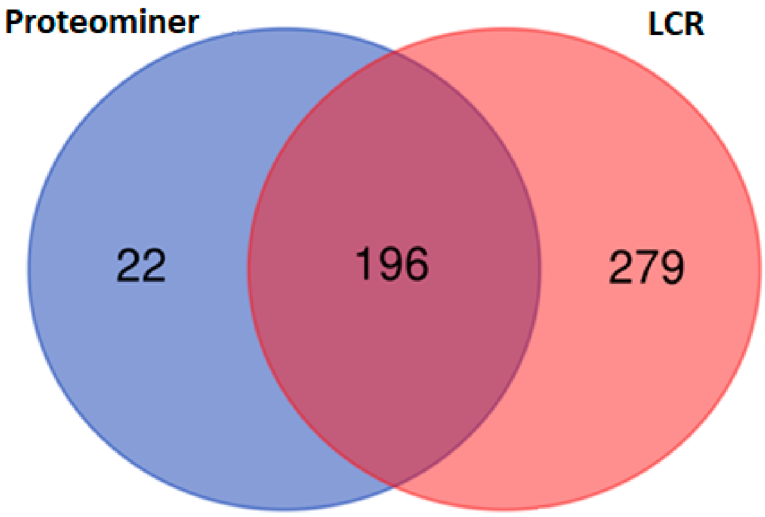

B

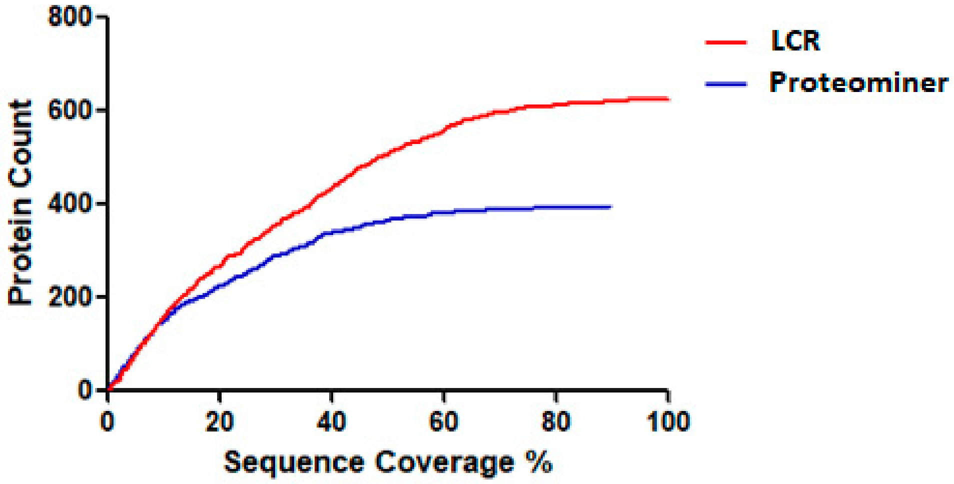

Supplementary Figure S3. A. Venn Diagram comparing proteins identified during LC-MS/MS analyses of serum samples (*n* = 12) prepared using the commercial Proteominer™ elution buffer and LCR. **B.** Cumulative frequency distribution graph comparing proteins detected in samples prepared using the commercial Proteominer™ elution buffer and LCR.

S4. Enzyme Linked Immunosorbent Assay analysis of Alpha-1-Acid Glycoprotein abundance change in YCW 1 v control sera

To investigate the observed fold change (Log_2 Fold Change = 2.12) detected for Alpha 1 Acid Glycoprotein (A1AGP) of GHP1 samples on Day 35, replicate chicken A1AGP enzyme-linked immunosorbent assay (ELISA) (ab157690) (abcam®) were conducted on pooled control and GHP1 samples from Day 35 (**Figure S4**). Three pooled serum samples from Day 35 of GHP1 and controls were tested for A1AGP concentration. Albeit that observed assay consistency was somewhat low, the data show a trend that indicated an increase in A1AGP levels identical to that indicated by LC-MS/MS analysis.

A.

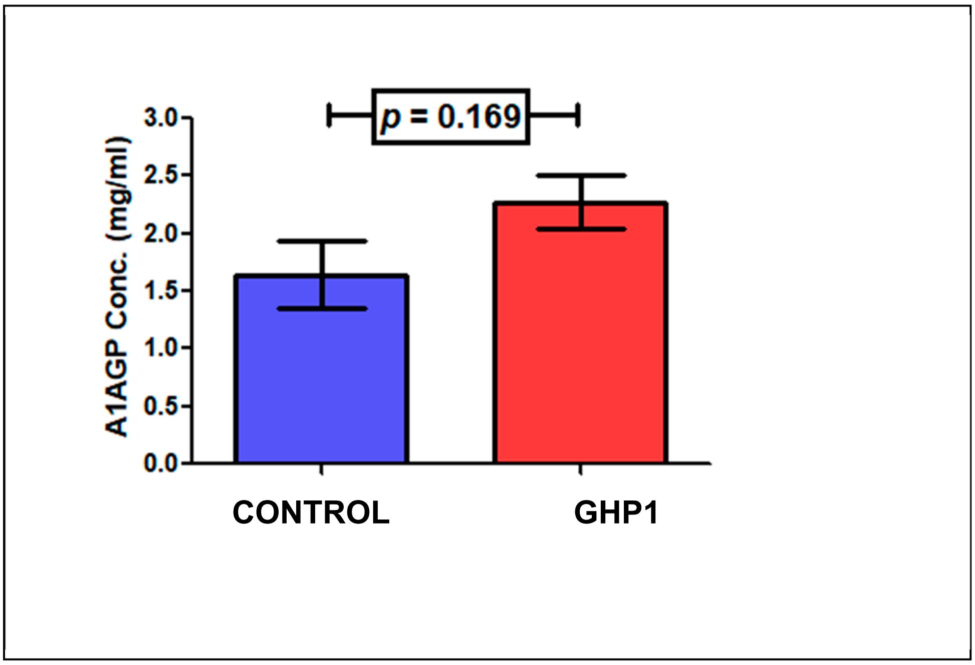

B.

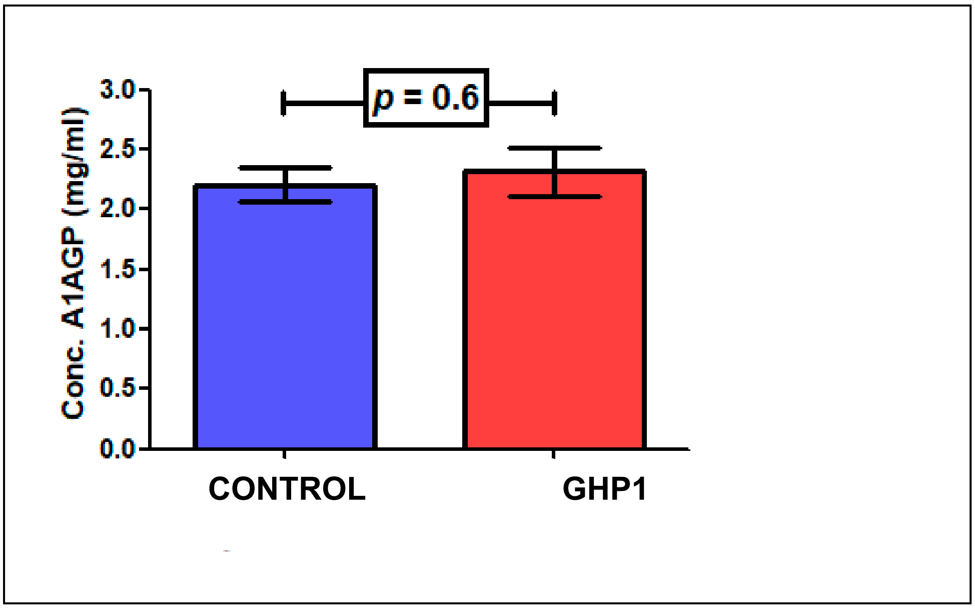

Supplementary Figure S4. ELISA confirmation of non-significant but detectable Alpha-1-Acid Glycoprotein (A1AGP) elevation in 35-day sera from GHP1 supplemented birds. **A.** A1AGP concentration in pooled serum samples from Control and GHP1-fed birds (ELISA analysis 1). **B.** A1AGP concentration in pooled serum samples from Control and GHP1-fed birds following the second ELISA analysis. Error bars denote standard error is shown.

Supplementary Table S1. Proteins of note approaching significance (0.16 > p > 0.08) in sera from **GHP1**-supplemented broilers.

Protein Description	Fold Change[1]	Peptides	Coverage (%)[2]	Day[3]	p value	Accession
Mannose-binding protein	0.414	8	32.7	Day 35	0.16	Q98TA4
Beta-hexosaminidase	0.27	20	54.1	Day 7	0.09	F1NTQ2
Beta-enolase	-1.2	12	41	Day 21	0.09	P07322
Alpha-amylase	0.8	19	61.7	Day 7	0.08	A0A1D5PUZ5

[1] Fold change refers to the $\log_2$ fold change in protein abundance in response to GHP1 treatment. [2] Coverage (%) refers to the % of protein sequence represented by identified peptides. [3] Day refers to the time point at which protein was detected: Day 7, Day 21 or Day 35 of feed trial. Proteins referred to in text are highlighted in (red).

Supplementary Table S2. Proteins approaching significance (p < 0.08) in sera from **GHP2**-supplemented broilers.

Protein Description	Fold Change[1]	Peptides	Coverage (%)[2]	Day[3]	p value	Accession
Transferrin receptor protein 1	2.01	10	18.3	Day 35	0.06	F1NTM6
Uncharacterized protein	1.36	6	45.3	Day 21	0.05	A0A1D5PUI7
Uncharacterized protein	0.86	6	16.8	Day 35	0.08	E1C206
Uncharacterized protein	0.86	82	63.0	Day 35	0.06	E1BRS7
Eukaryotic translation initiation factor 1	0.72	5	79.6	Day 21	0.06	R4GLE6
Eukaryotic initiation factor 4A-II	0.72	6	24.0	Day 7	0.06	R9PXN1
Alkaline phosphatase	0.71	16	42.8	Day 21	0.07	Q92058
Retinol-binding protein 4	0.66	14	70.9	Day 21	0.08	P41263
Uncharacterized protein	0.64	16	49.4	Day 21	0.06	E1BS56
T-complex protein 1 subunit zeta	0.64	12	36.2	Day 35	0.07	Q5ZJ54
Uncharacterized protein	0.54	2	14.8	Day 7	0.06	F1N9T1
Uncharacterized protein	0.53	32	70.9	Day 21	0.07	F1NVF3

Uncharacterized protein	0.40	8	45.5	Day 7	0.07	A0A1D5PAH2
Glutathione peroxidase	0.38	11	48.2	Day 7	0.06	F1NPJ8
Plasminogen	0.37	50	61.2	Day 21	0.08	F1NWX6
Collagen type XI alpha 1 chain	0.29	7	18.6	Day 21	0.06	A0A1D5PVT6
Uncharacterized protein	0.29	3	30.2	Day 7	0.08	F1N9A3
Uncharacterized protein	0.28	16	31.7	Day 21	0.07	F1P4N9
Apolipoprotein A-I	0.19	55	92.8	Day 21	0.07	P08250
G protein subunit beta 1	-0.19	4	18.2	Day 7	0.07	F1NLV4
Uncharacterized protein	-0.25	30	31.2	Day 7	0.07	A0A1D5PEF7
Fibulin-1	-0.26	18	38.5	Day 21	0.05	A0A1L1RU28
Uncharacterized protein	-0.38	4	52.9	Day 21	0.06	A0A1L1RML6
Uncharacterized protein	-0.39	6	35.7	Day 7	0.08	R4GFI8
MHC class II beta chain 2	-0.46	3	18.6	Day 21	0.06	A5HUL4
Cathepsin B	-0.48	17	60.9	Day 35	0.07	A0A1L1RS19
F-actin-capping protein subunit alpha-1	-0.50	6	33.9	Day 35	0.05	P13127
Chemokine	-0.64	4	48.9	Day 35	0.07	E1C733
Uncharacterized protein	-0.65	2	38.2	Day 7	0.07	F1NSD3
Elastin	-0.65	3	6.1	Day 35	0.06	P07916
Uncharacterized protein	-0.66	27	52.1	Day 35	0.07	F1NAB7
Histone H2A.J	-0.66	4	32.6	Day 7	0.05	P70082
Tubulin beta-7 chain	-0.69	12	46.4	Day 21	0.07	P09244
Myosin light polypeptide 6	-0.74	4	27.2	Day 35	0.06	P02607
Hemoglobin subunit beta	-0.79	9	76.2	Day 7	0.05	P02112
Lamin-A	-0.93	17	28.3	Day 7	0.08	P13648

[1] Fold change refers to the log_2 fold change in protein abundance in response to GHP2 treatment. [2] Coverage (%) refers to the % of protein sequence represented by identified peptides. [3] Day refers to the time point at which protein was detected: Day 7, Day 21 or Day 35 of feed trial. Proteins referred to in text are highlighted in (red).

Supplementary Table S3. Proteins with high fold change (Fold Change >1.8) in sera from **GHP2**-supplemented broilers.

Protein Description	Fold Change[1]	Peptides	Coverage (%)[2]	Day[3]	*p* value	Accession
Alpha-1-acid glycoprotein	3.69	7	36.0	Day 35	0.37	Q8JIG5
Uncharacterized protein	2.61	11	24.4	Day 35	0.31	E1C8N1
Neuronal-glial cell adhesion molecule	2.60	14	21.6	Day 35	0.18	Q03696
Transferrin receptor protein 1	2.01	10	18.3	Day 35	0.06	F1NTM6
Uncharacterized protein	1.98	25	63.0	Day 35	0.53	A0A1L1S0P1
Hemoglobin subunit beta	1.97	15	88.4	Day 35	0.19	P02112
Insulin like growth factor binding protein acid labile subunit	1.86	12	31.1	Day 35	0.17	F1NI07
Ovotransferrin	1.85	50	74.6	Day 35	0.32	A0A1D5P4L7
Hemopexin	1.82	23	81.7	Day 35	0.41	H9L385
Creatine kinase M-type	-1.81	19	51.4	Day 21	0.26	P00565

[1] Fold change refers to the $\log_2$ fold change in protein abundance in response to GHP2 treatment. [2] Coverage (%) refers to the % of protein sequence represented by identified peptides. [3] Day refers to the time point at which protein was detected: Day 7, Day 21 or Day 35 of feed trial. Proteins referred to in text are highlighted in (red).

Supplementary Table S4. Proteins of note approaching significance in sera from **GHP3**-supplemented broilers.

Protein Description	Fold Change[1]	Peptides	Coverage (%)[2]	Day[3]	*p* value	Accession
Uncharacterized protein	-0.57	3	24.7	Day 35	0.05	A0A1L1RUZ7
Uncharacterized protein	-0.59	5	24.1	Day 21	0.06	A0A1D5PGB2
Ubiquitin-40S ribosomal protein S27a	-0.64	3	28.2	Day 7	0.07	P79781
Uncharacterized protein	-0.64	7	37.3	Day 21	0.06	A0A1D5PKX1
Uncharacterized protein	-0.65	8	42.7	Day 35	0.07	A0A1D5P4K6
Uncharacterized protein	-0.71	18	53.6	Day 35	0.06	P09207

Collagen type XVIII alpha 1 chain	-0.72	18	60.1	Day 35	0.05	Q5ZLJ7
Matrilin-3	-0.75	13	32.5	Day 7	0.06	O42401
Complement C6	-0.77	17	26.5	Day 7	0.07	B8ZX71
Collagen alpha 1(VI) chain	-0.79	12	19.0	Day 7	0.07	A0A1D5PWN6
F-actin-capping protein subunit beta isoforms 1 and 2	-0.89	12	46.8	Day 35	0.06	A0A1D5P342
Glycerol-3-phosphate dehydrogenase	-0.93	12	51.6	Day 21	0.08	A0A1D5P1Y7
Myosin light polypeptide	-1.00	4	27.2	Day 35	0.07	P02607
Glycerol-3-phosphate dehydrogenase	-1.06	2	8.6	Day 35	0.06	Q9PUU8
Uncharacterized protein	1.34	10	48.7	Day 35	0.07	A0A1D5PW77

[1] Fold change refers to the $\log_2$ fold change in protein abundance in response to GHP3 treatment. [2] Coverage (%) refers to the % of protein sequence represented by identified peptides. [3] Day refers to the time point at which protein was detected: Day 7, Day 21 or Day 35 of feed trial.

Supplementary Table S5. Proteins of note approaching significance (0.16 > p > 0.08) in sera from **GHP3**-supplemented broilers.

Protein Description	Fold Change[1]	Peptides	Coverage (%)[2]	Day[3]	p value	Accession
Glutathione peroxidase	0.78	15	58.3	Day 35	0.09	F1NPJ8

[1]Fold change refers to the $\log_2$ fold change in protein abundance in response to GHP3 treatment. [2]Coverage (%) refers to the % of protein sequence represented by identified peptides. [3]Day refers to the time point at which Glutathione Peroxidase was detected: Day 7, Day 21 or Day 35 of feed trial.

Supplementary Table S6. Proteins with high fold change in sera from **GHP3**-supplemented broilers.

Protein Description	Fold Change[1]	Peptides	Coverage (%)[2]	Day[3]	p value	Accession
Creatine kinase M-type	3.12	19	51.4	Day 21	0.13	P00565
Hemoglobin subunit alpha-D	2.11	17	93.6	Day 21	0.41	P02001
Alpha-1-acid glycoprotein	1.86	7	36.0	Day 35	0.36	Q8JIG5

[1] Fold change refers to the $\log_2$ fold change in protein abundance in response to GHP3 treatment. [2] Coverage (%) refers to the % of protein sequence represented by identified peptides. [3] Day refers to the time point at which protein was detected: Day 7, Day 21 or Day 35 of feed trial.

Supplementary Table S7. List of known selenoproteins obtained from Liu et al. [1]

Glutathione peroxidase 1	Methionine sulfoxide reductase B	Selenoprotein H
Glutathione peroxidase 2	Selenophosphate Synthetase 1	Selenoprotein I
Glutathione peroxidase 3	Selenophosphate Synthetase 2	Selenoprotein M
Glutathione peroxidase 4	Selenoprotein 15	Selenoprotein N
Iodothyronine deiodinase 1	Thioredoxin reductase 1	Selenoprotein O
Iodothyronine deiodinase 2	Thioredoxin reductase 2	Selenoprotein U
Iodothyronine deiodinase 3	Thioredoxin reductase 3	Selenoprotein W

Supplementary Table S8. Proteins in different samples which were detected to have a SeMet/SeCys substitution.

A). Proteins in unsupplemented **control** samples which were detected to have a SeMet/SeCys substitution

Protein Description	Sample Pool	Coverage (%) [1]	Peptides	Substitution Site Position	Accession
SEC31 homolog B, COPII coat complex component	Day 7 Pen 7 Pool	3.6	1	4;5	E1BXC8

B). Proteins in **GHP3** samples which were detected to have a SeMet/SeCys substitution

Protein Description	Sample Pool	Coverage (%) [2]	Peptides	Substitution Site Position	Accession
Coatomer subunit alpha	Day 7 Pen 9 Pool	0.8	1	719;720	A0A1D5P185

Golgin A4	Day 7 Pen 9 Pool	1.2	2	1320	A0A1D5PNT3
Nuclear factor related to kappaB binding protein	Day 7 Pen 9 Pool	0.8	1	934	E1BZI6
Translocase of outer mitochondrial membrane 34	Day 35 Pen 2 Pool	5.4	2	307	F1P4X4

[1] Coverage (%) refers to the % of protein sequence represented in identified peptides. [2] Coverage (%) refers to the % of protein sequence represented by identified peptides.

Supplementary Table S9. Glutathione Peroxidase (F1NPJ8) Log_2 fold change between control and **GHP3** samples on Day 7, Day 21 & Day 35.

Day[1]	Fold Change[2]	*p*-value	Coverage (%)[3]	Peptides
Day 7	0.099	0.44	45.4	11
Day 21	0.36	0.08	56.0	14
Day 35	0.78	0.09	58.3	15

[1]Day refers to the time point at which Glutathione Peroxidase was detected: Day 7, Day 21 or Day 35 of feed trial.[2]Fold change refers to the log_2 fold change in protein abundance in response to GHP3 treatment. [3]Coverage (%) refers to the % of protein sequence represented by identified peptides.

References:

1. Liu, Q.; Yang, J.; Cai, J.; Luan, Y.; Sattar, H.; Liu, M.; Xu, S.; Zhang, Z. Analysis of the Interactions Between Thioredoxin and 20 Selenoproteins in Chicken. *Biological Trace Element Research* **2017**, *179*, 304-317.
